# Testosterone treatment is associated with reduced adipose tissue dysfunction and nonalcoholic fatty liver disease in obese hypogonadal men

**DOI:** 10.1007/s40618-020-01381-8

**Published:** 2020-08-08

**Authors:** E. Maseroli, P. Comeglio, C. Corno, I. Cellai, S. Filippi, T. Mello, A. Galli, E. Rapizzi, L. Presenti, M. C. Truglia, F. Lotti, E. Facchiano, B. Beltrame, M. Lucchese, F. Saad, G. Rastrelli, M. Maggi, L. Vignozzi

**Affiliations:** 1 Andrology, Women’s Endocrinology and Gender Incongruence Unit, Department of Experimental Clinical and Biomedical Sciences “Mario Serio”, University of Florence, Viale Pieraccini 6, 50134 Florence, Italy; 2 Interdepartmental Laboratory of Functional and Cellular Pharmacology of Reproduction, University of Florence, Viale Pieraccini 6, 50134 Florence, Italy; 3 Gastroenterology Unit, Department of Experimental Clinical and Biomedical Sciences “Mario Serio”, University of Florence, Viale Pieraccini 6, 50134 Florence, Italy; 4 Endocrinology Unit, Department of Experimental Clinical and Biomedical Sciences “Mario Serio”, University of Florence, Viale Pieraccini 6, 50134 Florence, Italy; 5 General, Bariatric and Metabolic Surgery Unit, Santa Maria Nuova Hospital, , Piazza Santa Maria Nuova, 1, 50122 Florence, Italy; 6 Medical Affairs, Bayer AG, Kaiser-Wilhelm-Allee 1, 51373 Leverkusen, Germany; 7 I.N.B.B. (Istituto Nazionale Biostrutture E Biosistemi), Viale delle Medaglie d’Oro 305, 00136 Rome, Italy

**Keywords:** Testosterone, Adipose tissue, Liver, NAFLD, Obesity, Hypogonadism

## Abstract

**Purpose:**

In both preclinical and clinical settings, testosterone treatment (TTh) of hypogonadism has shown beneficial effects on insulin sensitivity and visceral and liver fat accumulation. This prospective, observational study was aimed at assessing the change in markers of fat and liver functioning in obese men scheduled for bariatric surgery.

**Methods:**

Hypogonadal patients with consistent symptoms (*n* = 15) undergoing 27.63 ± 3.64 weeks of TTh were compared to untreated eugonadal (*n* = 17) or asymptomatic hypogonadal (*n* = 46) men. A cross-sectional analysis among the different groups was also performed, especially for data derived from liver and fat biopsies. Preadipocytes isolated from adipose tissue biopsies were used to evaluate insulin sensitivity, adipogenic potential and mitochondrial function. NAFLD was evaluated by triglyceride assay and by calculating NAFLD activity score in liver biopsies.

**Results:**

In TTh-hypogonadal men, histopathological NAFLD activity and steatosis scores, as well as liver triglyceride content were lower than in untreated-hypogonadal men and comparable to eugonadal ones. TTh was also associated with a favorable hepatic expression of lipid handling-related genes. In visceral adipose tissue and preadipocytes, TTh was associated with an increased expression of lipid catabolism and mitochondrial bio-functionality markers. Preadipocytes from TTh men also exhibited a healthier morpho-functional phenotype of mitochondria and higher insulin-sensitivity compared to untreated-hypogonadal ones.

**Conclusions:**

The present data suggest that TTh in severely obese, hypogonadal individuals induces metabolically healthier preadipocytes, improving insulin sensitivity, mitochondrial functioning and lipid handling. A potentially protective role for testosterone on the progression of NAFLD, improving hepatic steatosis and reducing intrahepatic triglyceride content, was also envisaged.

**Clinical Trial Registration:**

ClinicalTrials.gov Identifier: NCT02248467, September 25th 2014

## Introduction

Obesity, in particular abdominal obesity, is currently one of the greatest public health issues worldwide, owing to its high prevalence and substantial morbidity and mortality. The functional abnormalities of adipocytes, more than the excess of adipose mass itself, represent the major pathogenic link between obesity and non-transmittable chronic diseases, including type 2 diabetes, metabolic syndrome (MetS) and non-alcoholic fatty liver disease (NAFLD), which are all associated, to different degrees, with an increased risk of cardiovascular disease (CVD).

In obesity, excessive calorie intake finally often leads to an impaired insulin signaling, adipocyte hypertrophy, and lipid handling resistance [1–4]. In particular, the increased lipolysis from adipose tissue promotes hepatic insulin resistance by over-activation of hepatic gluconeogenesis and de novo lipid synthesis with a parallel deterioration of fatty acid oxidation. One of the primary mechanisms responsible for insulin resistance is the dysfunction of the main cellular organelles dedicated to metabolic control, i.e. mitochondria [5, 6]. Indeed, in adipocyte, dysfunctional mitochondria causes the spillover of lipids into non-adipose tissues, thus contributing to insulin resistance via lipotoxicity in other districts, including liver and vascular beds [7–10]. Therefore, these pathogenic mechanisms crucially link obesity to NAFLD and cardio-metabolic disorders [8, 11, 12]. Noteworthy, NAFLD is nowadays considered as a multisystem disease, affecting extra-hepatic organs and regulatory pathways, therefore amplifying the risk of type 2 diabetes mellitus (T2DM), cardiovascular (CVD) and cardiac diseases [13, 14].

Adding a layer of complexity, MetS and visceral obesity are also strongly associated with the onset of hypogonadotropic hypogonadism in males [15–17], due to functional abnormalities in the hypothalamus. Preclinical animal models demonstrated that MetS-induced hypogonadotropic hypogonadism is associated with a hypothalamic metainflammation, and with a substantial reduction of GnRH neurons [18, 19]. On the other hand, existing evidence consistently shows that testosterone therapy (TTh) induces a beneficial effect on metabolic parameters. In particular, several randomized [20–24] and observational [25, 26] trials demonstrated that TTh is able to improve body composition [27] and insulin sensitivity [20]. In an experimental rabbit model of high fat diet-induced MetS, our group previously demonstrated that in vivo T dosing prevented visceral adipose tissue (VAT) expansion [18, 28] and counteracted its derangements, normalizing preadipocyte maturation, lipid handling and insulin sensitivity [29]. In the same animal model, in vivo T dosing was also effective in counteracting all NAFLD features induced by MetS, such as lipid accumulation, inflammation and initial fibrosis within the liver [29]. This is in keeping with other preclinical studies in animal models [30]. Finally, corroborating preclinical evidence indicated that testosterone treatment exerts beneficial metabolic effects mainly by improving MetS-related liver and adipose tissue dysfunctions [29, 31]. In humans, the ameliorating effects of androgens on MetS-associated adipose tissue and liver disease is supported by limited data [32, 33]. Therefore, perhaps surprisingly, the mechanism underlying how TTh improves whole-metabolic control was still missing. To further ascertain the effect of TTh on visceral adipose tissue and liver in a human setting, we designed an observational, prospective study enrolling severely obese male subjects on a waiting list to undergo bariatric surgery and stratified them, at baseline, as having T above (eugonadal) or below (hypogonadal) the normal value. A subgroup of hypogonadal subjects that also presented sexual symptoms were treated with TTh, according to guidelines, during the period before the surgical procedure. Therefore, our study design allows us to investigate the effect of in vivo TTh on VAT and liver dysfunction in obese hypogonadal men undergoing surgery for weight loss, as compared to untreated hypogonadal and eugonadal subjects. The assessment of the main morpho-functional features of human VAT preadipocytes, isolated from the three different treatment groups, was then performed, through analysis of glucose uptake, mitochondrial morphology and mRNA expression. We also studied the effect of TTh by evaluating morphological alterations and mRNA expression profiles of major pathogenic pathways related to NAFLD. These results are part of an observational study aimed at evaluating the effect of TTh on different complications of obesity in a population of severely obese men, candidates for bariatric surgery for weight loss.

## Methods

Experimental procedures were carried out using the facilities of the Molecular Medicine Facility, Department of Biomedical Experimental and Clinical Sciences “Mario Serio”, University of Florence.

### Study design

This paper reports the results of the secondary analyses of an observational prospective study enrolling 78 severely obese men with indication for bariatric surgery. Table 1 reports the STROBE checklist showing compliance with guidelines. The study protocol was in accordance with the Declaration of Helsinki and was approved by the local ethics committee (protocol 2013/0006753; Careggi Hospital, Florence, Italy) and registered on the U.S. National Library of Medicine Registry (ClinicalTrials.gov Identifier: NCT02248467). Informed consent was obtained before initiation of any clinical procedure.

**Table 1:** STROBE statement—checklist

	Item no	Recommendation
Title and abstract	1	(a) Indicate the study’s design with a commonly used term in the title or the abstract The abstract describes the study design as “an observational, prospective study”
		(b) Provide in the abstract an informative and balanced summary of what was done and what was found The abstract describes the methods and findings
Introduction		
Background/rationale	2	Explain the scientific background and rationale for the investigation being reported The background and rationale are described in the Introduction, pages 3 & 4
Objectives	3	State specific objectives, including any prespecified hypotheses The specific aims of the study are stated in the last 2 paragraphs of the Introduction, page 4
Methods		
Study design	4	Present key elements of study design early in the paper The study design is discussed in the Introduction and in the “Study design and treatment” paragraph of the Methods section
Setting	5	Describe the setting, locations, and relevant dates, including periods of recruitment, exposure, follow-up, and data collection The institutional setting, study locations are described and study timing is discussed in the “Study design and treatment” paragraph of the Methods section
Participants	6	(*a*) Give the eligibility criteria, and the sources and methods of selection of participants. Describe methods of follow-up Selection of the sample is discussed in the “Study design and treatment” paragraph of the Methods section
		(*b*) For matched studies, give matching criteria and number of exposed and unexposed N/A
Variables	7	Clearly define all outcomes, exposures, predictors, potential confounders, and effect modifiers. Give diagnostic criteria, if applicable Outcomes, exposures, predictors etc. are discussed in the “Clinical parameters”, “Biochemical, metabolic and histological analyses”, “Isolation, characterization and differentiation of human visceral fat preadipocytes”, “Glucose uptake”, “RNA extraction and quantitative RT-PCR analysis”, “Fluorescence microscopy”, “Oxygen consumption analysis” and “Statistical analysis” paragraphs of the Methods section. The primary outcome of the clinical study was to evaluate the effect of TTh (Testosterone Therapy) on LUTS (Lower Urinary Tract Symptoms) in severely obese men with moderate to severe urinary symptoms, as evaluated by the International Prostate Symptom Score (IPSS) questionnaire; however, this and other secondary clinical outcomes are not reported in the manuscript and will be described in future publications. The outcomes reported in the present manuscript were changes over time between and within HYPO and HYPO + TTh groups in insulin sensitivity, adipogenic potential and mitochondrial function of preadipocytes (hPADs) isolated from adipose tissue biopsies and in the severity of NAFLD evaluated by triglycerides assay and liver biopsies histology
Data sources/measurement	8*	For each variable of interest, give sources of data and details of methods of assessment (measurement). Describe comparability of assessment methods if there is more than one group Measurement of the outcomes are discussed in the “Clinical parameters”, “Biochemical, metabolic and histological analyses”, “Isolation, characterization and differentiation of human visceral fat preadipocytes”, “Glucose uptake”, “RNA extraction and quantitative RT-PCR analysis”, “Fluorescence microscopy”, “Oxygen consumption analysis” and “Statistical analysis” paragraphs of the Methods section
Bias	9	Describe any efforts to address potential sources of bias The presence of a possible selection bias is reported in the Limitations
Study size	10	Explain how the study size was arrived at Sample determination is reported in the “Study design and treatment” paragraph of the Methods section. Sample size calculation was based on the difference in means for the primary outcome (change in IPSS total score) among 3 independent groups. For a two-sided paired Student’s t-test, with α and power equal to 5% and 90%, the enrollment of 30 patients per group was required to detect a minimum clinically significant difference between the changes of IPSS score measured at baseline and at follow-up
Quantitative variables	11	Explain how quantitative variables were handled in the analyses. If applicable, describe which groupings were chosen and why Use of variables is discussed in the “Statistical analysis” subsection
Statistical methods	12	(a) Describe all statistical methods, including those used to control for confounding
		(b) Describe any methods used to examine subgroups and interactions
		(c) Explain how missing data were addressed
		(d) If applicable, explain how loss to follow-up was addressed
		(e) Describe any sensitivity analyses Statistical methods are discussed in the “Statistical analysis” subsection
Results		
Participants	13*	(a) Report numbers of individuals at each stage of study—eg numbers potentially eligible, examined for eligibility, confirmed eligible, included in the study, completing follow-up, and analyzed
		(b) Give reasons for non-participation at each stage
		(c) Consider use of a flow diagram Numbers examined for eligibility, confirmed eligible, included in the study, completing follow-up, and analyzed are reported in the “Study design and treatment” subsection
Descriptive data	14*	(a) Give characteristics of study participants (eg demographic, clinical, social) and information on exposures and potential confounders
		(b) Indicate number of participants with missing data for each variable of interest
		(c) Summarize follow-up time (eg, average and total amount) Characteristics of study participants are reported in the “Characteristics of subjects undergoing bariatric surgery” subsection and in Table 2. Follow-up time is reported in the “Study design and treatment” subsection. There were no missing data for the outcomes considered in this manuscript
Outcome data	15*	Report numbers of outcome events or summary measures over time Measures are reported throughout the Results Section
Main results	16	(*a*) Give unadjusted estimates and, if applicable, confounder-adjusted estimates and their precision (eg, 95% confidence interval). Make clear which confounders were adjusted for and why they were included
		(*b*) Report category boundaries when continuous variables were categorized N/A
		(*c*) If relevant, consider translating estimates of relative risk into absolute risk for a meaningful time period N/A
Other analyses	17	Report other analyses done—eg analyses of subgroups and interactions, and sensitivity analyses N/A
Discussion		
Key results	18	Summarize key results with reference to study objectives Results are summarized in the Discussion section, page 17
Limitations	19	Discuss limitations of the study, taking into account sources of potential bias or imprecision. Discuss both direction and magnitude of any potential bias Limitations are discussed in the second-to-last paragraphs of the Discussion section (pages 21–22)
Interpretation	20	Give a cautious overall interpretation of results considering objectives, limitations, multiplicity of analyses, results from similar studies, and other relevant evidence See final paragraph of the Discussion section
Generalisability	21	Discuss the generalisability (external validity) of the study results See limitations in the second-to-last paragraphs of the Discussion section (pages 21–22)
Other information		
Funding	22	Give the source of funding and the role of the funders for the present study and, if applicable, for the original study on which the present article is based Funding is reported in the Title Page. The original study on which the present article is based is registered and described in the U.S. National Library of Medicine Registry (ClinicalTrials.gov Identifier: NCT02248467)

^*^Give information separately for exposed and unexposed groups

The primary outcome of the clinical study was to evaluate the effect of TTh on Lower Urinary Tract Symptoms (LUTS) in severely obese men with moderate to severe urinary symptoms, as evaluated by the International Prostate Symptom Score (IPSS) questionnaire [34]. Sample size calculation was based on the difference in means for the primary outcome among 3 independent groups and required the enrollment of 30 patients per group. The results of the primary outcome are not reported in this manuscript and will be described in future publications. The analysis of the effect of TTh on preadipocytes isolated from visceral adipose tissue and on histomorphometric and molecular parameters of liver tissue had been planned as secondary outcomes and are reported in this paper.

### Patient recruitment

**Fig. 1:**
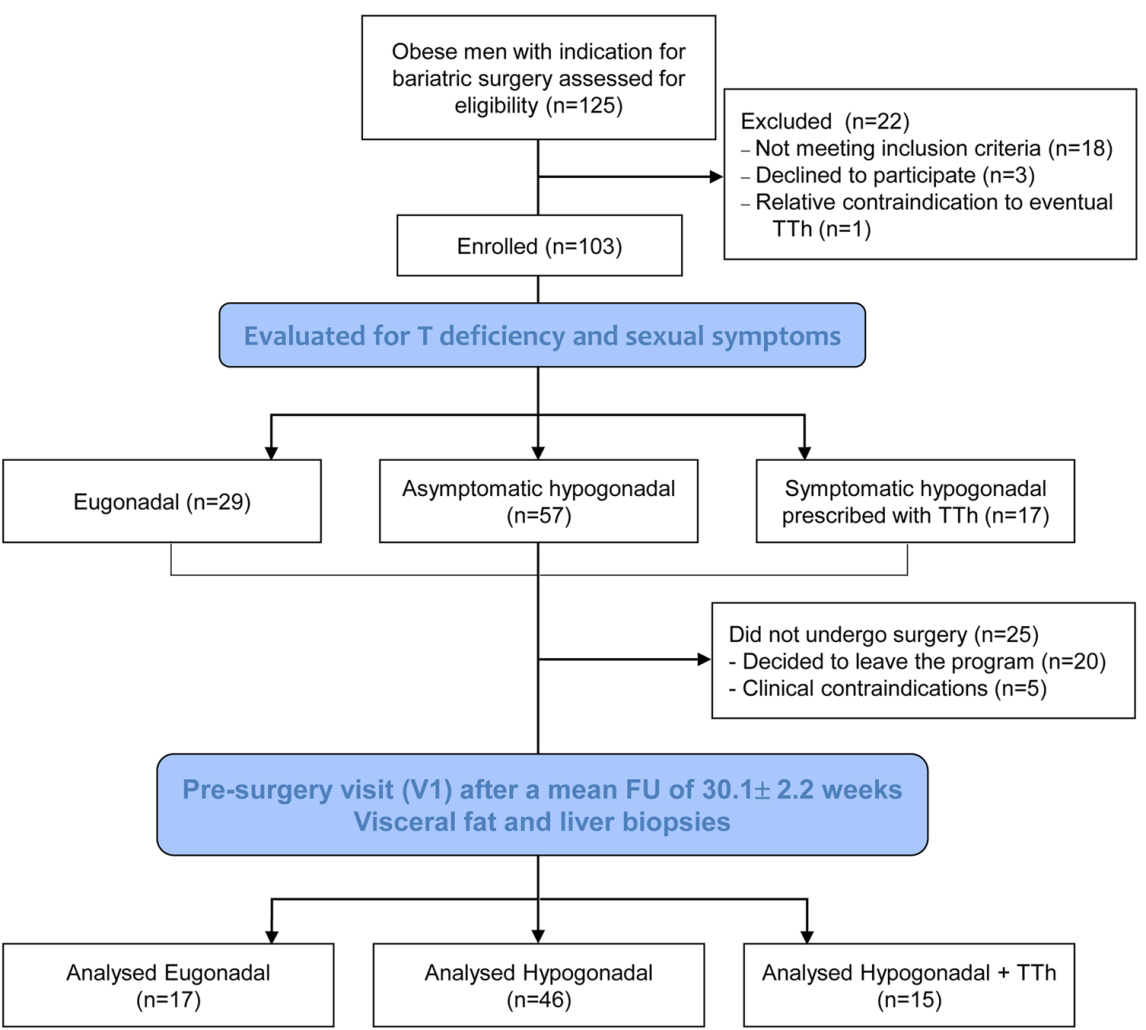
Flow-chart for the observational study. *T *testosterone, *TTh *testosterone therapy, *FU *follow-up

Patients with a recommendation for surgical treatment of obesity were initially screened at the General, Bariatric and Metabolic Surgery Unit, Santa Maria Nuova Hospital, Florence, Italy. Among the 125 patients screened, 103 were confirmed eligible and included; reasons for screening failure are reported in the study flow-chart (Fig. 1).

Eligible subjects were then informed about the study and those agreeing to participate were sent to the Sexual Medicine and Andrology Unit of the Careggi University Hospital (University of Florence) where the procedures pertaining to the baseline visit (V0) were performed from May 2013 to December 2016.

### Inclusion and exclusion criteria

All the following were considered as inclusion criteria for the present study: male subjects aged between 25 and 65 years; candidate for a bariatric surgery [body mass index (BMI) ≥ 40 kg/m^2^, or BMI is > 35 kg/m^2^ with obesity-related comorbidities, such as type 2 diabetes mellitus, hypertension and cardiovascular disease]. LUTS were defined by IPSS total score ≥ 8. All the following were considered as exclusion criteria: bladder failure or neurogenic bladder; post-void residual urine volume > 250 mL; multiple sclerosis, Parkinson’s disease, spinal cord injury; urethral stenosis, diverticula; history of prostatectomy, bladder neck surgery, transurethral resection of the prostate (TURP); severe systemic disease, including previous or active cancer; alcohol or drug abuse; uncontrolled psychiatric diseases.

### Interventions

According to the recommendations of the International Society of Andrology (ISA), the ISSAM and the European Association of Urology (EAU) [35], hypogonadism was defined as levels of total testosterone (*T*) < 12 nmol/L and/or free *T* < 225 pmol/L, calculated according to Vermeulen’s formula [36]. Based on the same recommendations, in line with the observational nature of the study, the decision of starting TTh was made in men with low *T* (total or free) and symptoms consistent with *T* deficiency, in particular sexual symptoms, which are considered to be the most specific to the condition [37]. Therefore, patients were defined as symptomatic when they reported the presence of all the symptoms explored by the 5 questions composing the sexual sub-score of the Aging Males’ Symptoms (AMS) scale [38] (see 2.6, “Clinical parameters”—feeling to have passed one’s peak, decrease in beard growth, impaired sexual potency, less morning erections, disturbed libido), thus obtaining a score of at least 10 in the sub-score.

Hence, enrolled patients were divided into three groups: eugonadal (EUG; *n* = 29), untreated hypogonadal (HYPO; *n* = 57) and symptomatic hypogonadal subjects (HYPO + TTh; *n* = 17). Symptomatic hypogonadal obese men received testosterone undecanoate (1000 mg i.m.) every 12 weeks after loading (second dose after 6 weeks). The treatment was prescribed to symptomatic men as soon as the results for total and calculated free T became available, usually within one week from V0. The choice of using this preparation has been made because injectable preparations, in contrast with transdermal ones, are characterized by a lower variability in the absorption [39]. The variability in transdermal absorption is mainly determined by the subcutaneous adipose tissue representation [39] and, therefore, we considered transdermal *T* preparation as unsuitable for severely obese men. Among the injectable preparations, long acting *T* undecanoate is the least likely to produce side effects, due to the stable plasmatic *T* concentrations achieved after its injection and maintained for the 12-week period [39].

### Follow-up

No drop-outs were registered during the study. However, 25 patients did not undergo bariatric surgery because they decided to leave the program or because no-TTh related clinical contraindications arose (Fig. 1). All the patients that actually underwent surgery (*n* = 78) completed the follow-up visit (V1) immediately before, providing data for the analyses reported in this article. Of these, *n* = 17 were eugonadal (EUG), *n* = 46 were untreated hypogonadal (HYPO) and *n* = 15 were treated symptomatic hypogonadal (HYPO + TTh; *n* = 15). The mean length of follow up was 30.1 ± 2.2 weeks.

During bariatric surgery, samples of liver and adipose tissue were taken, after a specific informed consent, for further analyses. Preadipocyte cell cultures (hPADs) were isolated from the visceral tissue specimens, whereas liver biopsies underwent histological examination for NAFLD scoring.

### Clinical parameters

At baseline (V0) and follow-up visit (V1), a physical examination was performed with measurements of body weight, height, BMI and waist circumference. BPH/LUTS symptoms were assessed by IPSS [34]. All patients were also asked to complete the Heinemann’s Aging Males’ Symptoms (AMS) scale [38] in its validated Italian version [40]. The AMS is a self-reported scale developed to quantify health-related quality of life and symptoms of aging men and is useful in both the diagnosis of hypogonadism and the monitoring of patients using testosterone therapy [41]. The AMS scale encompasses 17 questions with three sub-scales, including mind-, body- and sexual-related symptoms. Answers are codified with a 1–5 range (no symptom to extremely severe), and the total sum of all sub-scales provides a total score. A subset of patients underwent transrectal color-Doppler ultrasonography (CDUS) for the study of the prostate, according to previously published procedures [42], to assess LUTS-related secondary outcomes not reported in this paper.

### Biochemical, metabolic and histological analyses

Blood samples for biochemical and metabolic parameters were obtained from patients at baseline (V0) and at follow-up visit (V1). In particular, blood samples were drawn in the morning after an overnight fast to determine total testosterone (chemiluminescence immunoassay; Advia Centaur, Siemens, Berlin, Germany), sex hormone binding globulin (SHBG; electro-chemiluminescence immunoassay; COBAS 600, Roche Diagnostics, Basel, Switzerland); 17β-estradiol (luminescent oxygen channeling immunoassay; Vista, Siemens, Berlin, Germany); blood glucose (glucose hexokinase method; Dimension Vista 1500, Medical Solutions Siemens Healthcare, Malvern, PA); total cholesterol, high-density lipoprotein cholesterol (HDL), and triglycerides (automated enzymatic colorimetric method; Dimension Vista 1500, Medical Solutions Siemens Healthcare); insulin (electro-chemiluminescence immunoassay; Roche Diagnostics, Mannheim, Germany); aspartate aminotransferase (AST), alanine aminotransferase (ALT) and gammaglutamyl-transferase (γGT) (LOCI® luminescent oxygen channeling immunoassay, advanced homogeneous chemiluminescence; Dimension Vista 1500, Medical Solutions Siemens Healthcare); low-density lipoprotein cholesterol was estimated indirectly using the Friedewald equation [low-density lipoprotein cholesterol = total cholesterol / (high-density lipoprotein cholesterol + triglycerides/5)], where all parameters are expressed in milligrams per deciliter.

Total and/or free testosterone measured at baseline (V0) were used to categorize patients into the 3 analytical groups (see above).

The fatty liver index (FLI), and index of hepatic steatosis, was calculated as: FLI = [e 0.953*loge (triglycerides) + 0.139*BMI + 0.718*loge (GGT) + 0.053*waist circumference—15.745]/[1 + e 0.953*loge (triglycerides) + 0.139*BMI + 0.718*loge (GGT) + 0.053*waist circumference—15.745] * 100, as in Bedogni et al. [43]. Calculated free testosterone was derived according to the formula of Vermeulen and collaborators (available at https://www.issam.ch/freetesto.htm) [36]. Triglyceride content from liver biopsies was evaluated using the Triglyceride Quantification Colorimetric/Fluorometric Kit (BioVision, Milpitas, CA), following the manufacturer’s instructions.

Liver biopsies were processed routinely for paraffin embedding, and 3 micron-thick sections were prepared for histological analysis, as previously described [29]. For each sample, three to five sections were cut along the length. Hematoxylin and eosin-stained sections were used for direct microscopic examination, using the Nikon Microphot-FXA microscope (Nikon, Tokyo, Japan).

### Isolation, characterization and differentiation of human visceral fat preadipocytes

Human visceral fat preadipocytes (hPADs) isolation was performed, as previously described [8]. After dissection under sterile conditions, visceral adipose tissue (VAT) samples were immediately placed in serum-free (SF) DMEM/F12, supplemented with 200 μg/mL streptomycin and 200 U/mL penicillin, and then digested with type 2 collagenase (1 mg/mL in PBS) for 1 h at 37 °C. After filtration and centrifugation the cells pellet was plated onto 100-mm cell culture dishes and cultured, at 37 °C in humidified atmosphere of 95% air-5% CO_2_, in complete culture medium (DMEM containing 10% FBS, 100 μg/mL streptomycin, 100 U/mL penicillin, 2 mM l-glutamine, and 1 μg/mL amphotericin-B).

Conjugated monoclonal antibodies CD34-FITC, CD45-FITC, CD31-PE, CD14-PE, CD90-FITC, CD106-PE and CD105-PE were used to characterize hPADs at P1 by flow cytometry. hPADs isolated from the different groups showed a similar immunophenotypic profile, characterized by mesenchymal stem cell markers (CD90, CD105, and CD106). Moreover, all hPADs were negative for endothelial (CD31), hematopoietic (CD34 and CD45), and monocytic (CD14) markers.

The hPADs differentiation, 2 days after confluence (time 0), was induced by exposing cells to a differentiation mixture (DIM) containing 5 mg/mL insulin, 1 mM dexamethasone, and 0.5 mM 3-isobutyl-1-methylxanthine (IBMX) in 5% stripped FBS-supplemented DMEM for 8 days. The culture medium was replaced every 48 h, and then the cells were shifted to a medium containing 5 mg/mL insulin for 48 h.

### RNA extraction and quantitative RT-PCR analysis

Isolation of total RNA from tissues and hPADs was performed using TRIzol reagent (Life Technologies, Paisley, UK) and/or Qiagen RNeasy Mini Kit (Qiagen, Hilden, Germany), in accordance with the manufacturer’s instruction. cDNA synthesis and real-time RT-PCR experiments were performed using reagents and instrumentation from Bio-Rad Laboratories (Hercules, CA), as previously described [44]. Specific PCR oligonucleotides were based on sequences obtained from Ensemble Genome (https://www.ensembl.org) and NCBI GenBank (https://www.ncbi.nlm.nih.gov). The 18S ribosomal RNA subunit was used as the reference gene for the relative quantization of the target genes based on the comparative threshold cycle (Ct) 2^−ΔΔCt^ method [45].

### Glucose uptake

Glucose uptake in DIM-hPADs was performed as follows: hPADs were shifted in SF medium for 24 h, followed by increasing concentrations of insulin (1, 5, 10, and 50 nM for 30 min) diluted in glucose-free Krebs phosphate buffer (2.5 mmol Ca^2+^, 1 mg/mL BSA), to evaluate insulin-dependent stimulation. After insulin incubation, the cells were further incubated with ^3^H-2-deoxy-d-glucose [16 mM (1 mCi/mL)] for 10 min. Cells were then washed with PBS, lysed with NaOH 0.5 M, and incorporated radioactivity was measured by scintillation spectrometry using a β-counter.

### Fluorescence microscopy

To evaluate the mitochondrial network and dynamics DIM-induced hPADs isolated from each experimental group, were cultured on 35 mm u-Dish (Ibidi GmbH, Munich, Germany) for 10 days and then stained with 200 nM MitoTracker Green^FM^ (Invitrogen Life Technologies, Carlsbad, CA), as previously described [8]. Cells were imaged, with time lapses recorded for 3 min with time intervals of 10 s, using an inverted Leica DMI6000 microscope (Leica, Wetzlar, Germany) equipped with a stage incubator (Pecon, Erbach, Germany) with controlled temperature, humidity and CO_2_.

Superoxide radical production in hPADs was monitored by dihydroethidium (DHE; Invitrogen) fluorescence and quantified by measuring the change in fluorescence intensity in the nuclei of rPAD cells during imaging, as previously described [8].

Images were captured through a 63 × 1.2NA water immersion objective, with a DFC350FX camera and Leica filter set L5 (for MitoTracker Green), and N2.1 and N3 (for DHE). All image analyses were performed using Fiji ImageJ software [46].

### Oxygen consumption analysis

Quantification of oxygen consumption by hPADs isolated from each experimental group was conducted by means of the Oxygraph system (Hansatech Instruments, Pentney, UK), as previously described [47]. Briefly, cells (7.5 × 10^4^) were loaded in the chamber, which contained 300 μL of DMEM with glutamine 2 mM and sodium succinate 20 mM. Oxygen consumption was monitored for 5 min at 37 °C.

### Statistical analysis

Results are expressed as mean ± SEM, when normally distributed, and median [interquartile range] and SEM when non-normally distributed.

Differences between EUG, HYPO and HYPO + TTh in molecular and morphological parameters were assessed by Kruskal–Wallis test adjusted for multiple comparisons followed by Mann–Whitney test where appropriate to evaluate differences between the groups, with *p* < 0.05 considered as significant. Correlations were assessed using Spearman's method, and the statistical analysis was performed with the Statistical Package for the Social Sciences for Windows (SPSS v.26.0; SPSS Inc., Chicago, IL). Half-maximal response effective concentration (EC_50_) values and maximal effect (*E*_max_) values were calculated using the computer program ALLFIT [48].

Changes over time between and within HYPO and HYPO + TTh groups were assessed by the multilevel mixed-effects linear regression, which takes into account the effect of time and treatment and their interaction. These data were adjusted for the baseline value of the outcome variable and for age and baseline BMI. These analyses were conducted using Stata MP 13.1 for Windows (Stata Corp, College Station, TX).

For the aforementioned analyses, which evaluated changes over time in clinical, biochemical and instrumental parameters, HYPO was considered as the comparator group. For the analyses performed on samples of adipose or liver tissue, which do not have a baseline and follow-up evaluation, EUG was considered as the comparator.

## Results

### Characteristics of subjects undergoing bariatric surgery at baseline and at surgery

Baseline features of the three study arms are reported in Table 2. No baseline differences between the three groups were observed, with the exception of total (TT), SHBG and calculated free testosterone (cFT) levels. As expected, TT and cFT were significantly reduced (*p* < 0.0001) in the two hypogonadal groups (untreated and testosterone-treated), as compared to eugonadal subjects (EUG: TT = 15.7 ± 0.9 nmol/L, cFT = 311.2 ± 14.1 pmol/L; HYPO: TT = 7.6 ± 0.4 nmol/L, cFT = 157.1 ± 6.4 pmol/L; HYPO + TTh: TT = 7.4 ± 0.5 nmol/L, cFT = 153.0 ± 9.4 pmol/L). Furthermore, consistent with the rationale for treatment, AMS sexual symptoms score at baseline was significantly higher in HYPO + TTh [12.0 (11.0–15.0)] than in EUG [6.5 (5.0–13.2)] and HYPO [7.0 (5.0–10.5)] subjects (both differences *p* < 0.0001).

**Table 2:** Baseline characteristics of enrolled patients and differences among the three groups

	Eugonadal (*n* = 17)	Hypogonadal (*n* = 46)	Hypogonadal + TTh (*n* = 15)
Age (years)	44.5 ± 3.1	44.7 ± 1.4	50.1 ± 2.8
BMI (kg/m^2^)	42.4 ± 1.8	47.2 ± 1.3	42.5 ± 1.2
WC (cm)	133.7 ± 4.0	146.1 ± 2.4	131.7 ± 2.8
Systolic BP (mmHg)	126.1 ± 3.5	133.5 ± 2.7	131.7 ± 2.8
Diastolic BP (mmHg)	77.3 ± 2.2	83.6 ± 1.7	83.0 ± 2.3
Use of on demand PDE5i % (*n*)	(0)	(0)	(2)
Use of glucose-lowering drugs % (*n*)	23.5 (4)	26.1 (12)	20.0 (3)
Metformin % (*n*)	(4)	(12)	(3)
Repaglinide % (*n*)	(0)	(2)	(1)
GLP-1 analogs % (*n*)	(1)	(0)	(2)
DPPIV inhibitors % (*n*)	(0)	(2)	(1)
Insulin % (*n*)	(0)	(2)	(0)
Use of lipid-lowering drugs (%)	5.9 (1)	13.0 (6)	20.0 (3)
Statin (%)	(1)	(4)	(3)
Fibrates (%)	(0)	(2)	(0)
Omega-3 fatty acids (%)	(0)	(1)	(0)
Ezetimibe (%)	(0)	(1)	(0)
Total testosterone (nmol/l)	15.7 ± 0.9	7.6 ± 0.4*	7.4 ± 0.5*
SHBG (nmol/l)	38.2 [34.6–46.9]	**25.0 [14.6–34.9]°**	**32.3 [18.6–34.5]°**
cfT (pmol/l)	311.2 ± 14.1	157.1 ± 6.4*	153.0 ± 9.4*
LH (U/l)	5.6 [3.4–6.7]	3.8 [2.7–5.4]	3.2 [1.8–5.1]
17β estradiol (pmol/l)	153.5 [104.2–180.0]	130.0 [95.0–150.0]	101.0 [85.0–140.0]
Hematocrit (%)	44.5 ± 1.3	45.1 ± 0.5	44.5 ± 1.2
PSA (ng/dl)	0.5 [0.3–0.7]	0.5 [0.3–0.9]	0.5 [0.4–1.3]
Glycaemia (g/l)	1.1 ± 0.1	1.2 ± 0.1	1.2 ± 0.1
HbA1c (mmol/mol)	40.2 ± 2.2	45.4 ± 2.6	46.3 ± 3.7
Triglycerides (mg/dl)	97.0 [74.0–188.0]	145.0 [110.0–205.5]	152.0 [105.0–236.0]
Total cholesterol (mg/dl)	187.6 ± 9.8	208.3 ± 8.3	199.4 ± 10.7
HDL cholesterol (mg/dl)	44.1 ± 2.4	42.6 ± 1.7	39.0 ± 1.5
LDL cholesterol (mg/dl)	115.0 ± 5.7	133.5 ± 7.1	118.9 ± 9.6
T2DM % (*n*)	(4)	(9)	(3)
Hypertension % (*n*)	(7)	(20)	(9)
Cardiovascular diseases % (*n*)	(4)	(4)	(2)
MetS % (*n*)	61.5 (8)	87.5 (35)	80 (12)
AMS total score	30.5 [26.0–34.5]	32.5 [25.5–39.5]	32.0 [26.0–40.0]
AMS sexual symptoms score	6.5 [5.0–13.2]	7.0 [5.0–10.5]	12.0 [11.0–15.0]*
IPSS total score	10 [8–25]	8 [8–24]	10 [8–16]

Data are expressed as mean ± SEM when normally distributed and as percentages when categorical. Bold value indicates statistical significance difference. *p < 0.0001, °*p* < 0.05 vs. Eugonadal

*TTh* testosterone therapy, *BMI* body mass index, *WC* waist circumference, *BP* blood pressure, *PDE5i* phosphodiesterase type 5 inhibitors, *GLP* glucagon-like peptide-1, *DPPIV* dipeptidyl peptidase 4, *SHBG* sex hormone binding globulin, *cFT* calculated free testosterone, *LH* luteinizing hormone, *PSA* prostate specific antigen, *HbA1c* glycated hemoglobin, *HDL* high-density lipoprotein, *LDL* low-density lipoprotein, *T2DM* type 2 Diabetes Mellitus, *MetS* Metabolic Syndrome, *AMS* Aging Males’ Symptoms scale, *IPSS* International Prostatic Symptoms Score

Mean duration of follow-up (33.14 ± 6.78, 30.28 ± 2.84 and 27.63 ± 3.64 weeks in EUG, HYPO and HYPO + TTh, respectively) was not significantly different among the three groups (*p* = 0.863). Similarly, at V1, no differences were highlighted in the percentage of patients using glucose-lowering [23.5 (*n* = 4), 28.3 (*n* = 13) and 26.7% (*n* = 4), in EUG, HYPO and HYPO + TTh, respectively; *p* = 0.913] or lipid-lowering drugs [5.9 (*n* = 1), 21.7 (*n* = 10) and 20.0% (*n* = 3) in EUG, HYPO and HYPO + TTh, respectively; *p* = 0.324], and in the percentage of subjects following a pre-surgery diet plan [5.9 (*n* = 1), 13.0 (*n* = 6) and 6.7% (*n* = 1), in EUG, HYPO and HYPO + TTh, respectively; *p* = 0.621] among the three groups. Of note, no specific medications for obesity (i.e. naltrexone-bupropion or liraglutide) were prescribed between baseline and V1.

### Biochemical characteristics of hypogonadal men and their change over time according to TTh

**Fig. 2:**
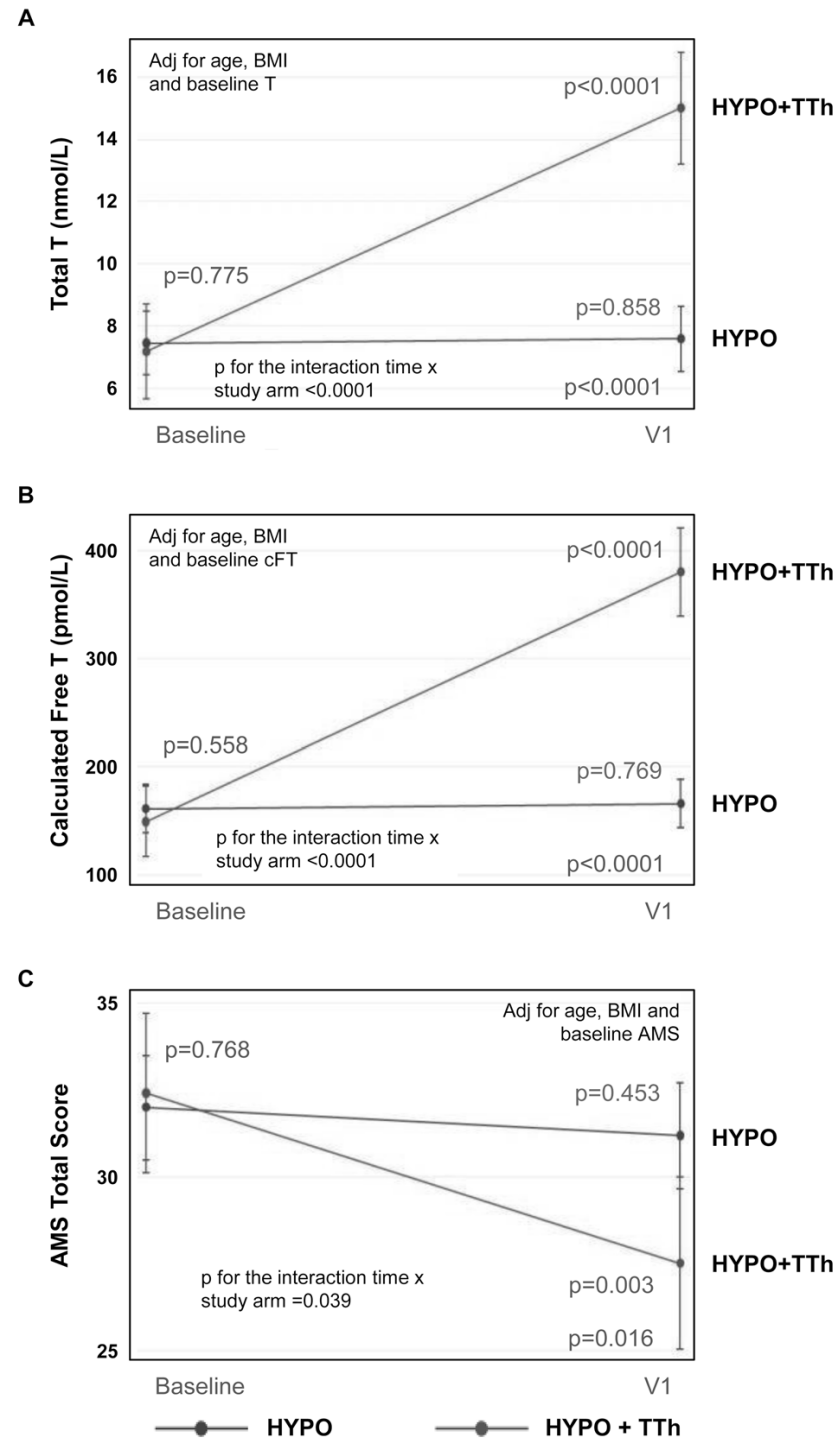
Variation of biochemical and clinical parameters relative to hypoandrogenism in hypogonadal (HYPO) vs. hypogonadal subjects treated with testosterone (HYPO + TTh), V1 (surgery) vs. baseline. Data were adjusted for the baseline value of the outcome variable and for age and baseline BMI. *AMS *Aging Males’ Symptoms scale, *BMI *body mass index, *cFT *calculated free testosterone, *T *testosterone, *TTh *testosterone therapy

Considering the longitudinal data, significant changes in insulin (*p* < 0.0001), triglycerides (*p* = 0.010), AST (*p* = 0.002) and ALT (*p* = 0.001) serum levels were observed between baseline and V1 in the untreated-hypogonadal patients group, but not in the TTh-treated group (Table 3). TTh-hypogonadal patients showed a significant reduction of total cholesterol and fatty liver index at the V1 pre-surgery visit, as compared to baseline (*p* = 0.022 and *p* = 0.001, respectively), whereas in the untreated group we observed no significant changes (Table 3). Figure 2 shows total (TT) and circulating free T (cFT) levels in these two study arms. As expected, both TT and cFT significantly increased over time in the TTh arm, but no change occurred in the untreated-hypogonadal group (Fig. 2 panels a, b; *p* < 0.0001 for the interaction time ×treatment for both TT and cFT). Accordingly, the Aging Males' Symptom (AMS) scale total score significantly improved in the T-treated hypogonadal patients (*p* < 0.01 vs. baseline), but not in the untreated-hypogonadal group (Fig. 2 panel c; *p* = 0.039 for the interaction time × treatment). The differences at V1 between untreated and testosterone-treated hypogonadal patients were significant (*p* < 0.0001 for both TT and cFT, and *p* < 0.05 for AMS) (Fig. 2 panels a–c).

**Table 3:** Characteristics of hypogonadal untreated (HYPO) and testosterone-treated (HYPO + TTh) patients at baseline and before surgery (V1)

Variable	Treatment	Baseline	V1	V1 vs. baseline within group (*p*)
BMI (kg/m^2^)	HYPO HYPO + TTh	46.6 [43.7–49.3] 46.1 [41.6–50.7]	47.6 [44.6–50.6] 45.5 [41.0–50.0]	0.619 0.848
	Interaction time × treatment *p* = 0.665			
Fasting glucose (mg/dL)	HYPO HYPO + TTh	119.8 [106.6–133.0] 123.3 [104.1–142.3]	138.6 [124.9–152.4] 122.8 [100.2–145.4]	**0.050** 0.979
	Interaction time × treatment *p* = 0.275			
Insulin (mU/L)	HYPO HYPO + TTh	39.2 [31.1–47.2] 36.0 [24.1–48.0]	16.6 [8.2–25.1] 21.3 [6.0–36.6]	**< 0.0001** 0.130
	Interaction time × treatment *p* = 0.494			
Total cholesterol (mg/dL)	HYPO HYPO + TTh	201.8 [191.7–211.8] 204.5 [189.4–219.6]	201.1 [190.7–211.5] 177.8 [159.9–195.6] *	0.927 **0.022**
	Interaction time × treatment *p* = 0.059			
HDL cholesterol (mg/dL)	HYPO HYPO + TTh	41.0 [38.7–43.2] 41.2 [37.8–44.6]	38.7 [36.3–41.0] 34.6 [30.6–38.6]	0.154 **0.011**
	Interaction time × treatment *p* = 0.165			
Triglycerides (mg/dL)	HYPO HYPO + TTh	153.5 [133.9–176.0] 143.3 [116.7–175.9]	197.3 [171.2–227.3] 160.9 [126.1–205.4]	**0.010** 0.456
	Interaction time × treatment *p* = 0.464			
Aspartate aminotransferase (AST; U/L)	HYPO HYPO + TTh	28.4 [20.6–36.2] 31.1 [19.6–42.5]	45.3 [37.4–53.2] 38.7 [24.7–52.7]	**0.002** 0.386
	Interaction time × treatment *p* = 0.373			
Alanine aminotransferase (ALT; U/L)	HYPO HYPO + TTh	45.0 [35.2–54.9] 50.6 [36.1–65.0]	67.9 [57.8–77.9] 50.0 [32.4–67.7]	**0.001** 0.960
	Interaction time × treatment *p* = 0.076			
Fatty Liver Index	HYPO HYPO + TTh	99.1 [98.8–99.4] 99.2 [98.8–99.7]	99.1 [98.8–99.3] 98.4 [97.9–98.8] *	0.797 **0.001**
	Interaction time × treatment *p* = 0.007			

Data are expressed as mean with range in brackets. Data were adjusted for the baseline value of the outcome variable and for age and baseline BMI. Significant differences are in bold

*p < 0.001 vs Hypogonadal

### Correlations between liver biopsies scores, TTh and cFT levels and liver triglycerides levels at time of surgery

**Fig. 3:**
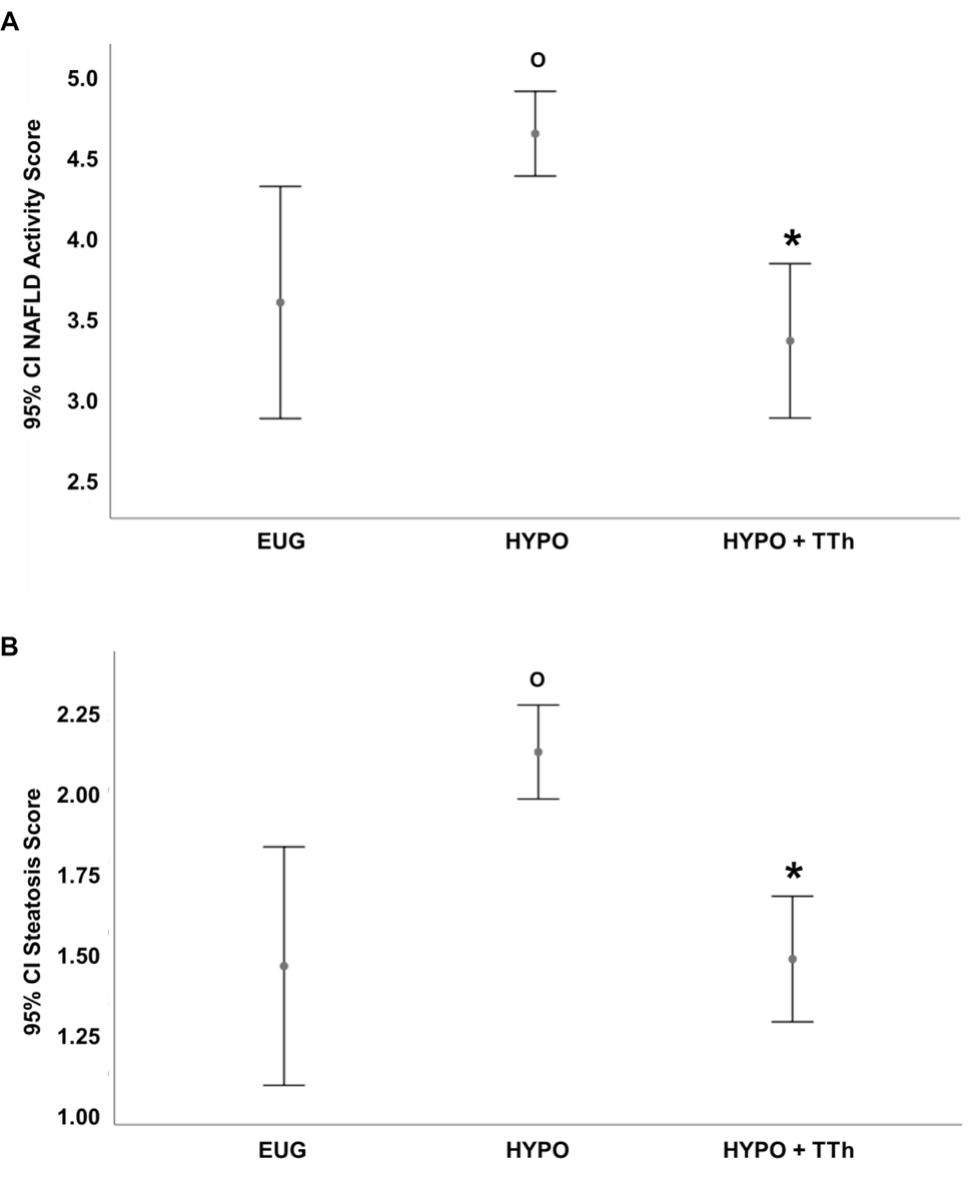
Nonalcoholic Fatty Liver Disease (NAFLD) Activity Score (NAS) (**a**) and Steatosis Score (**b**) derived from liver biopsies according to the three experimental groups. ^**O**^*p* < 0.05 vs. Eugonadal; *p < 0.05 vs. Hypogonadal

Figure 3 reports liver NAFLD activity score (NAS) and steatosis score according to the three experimental groups (eugonadal, untreated hypogonadal and T-treated hypogonadal). NAS score was significantly higher in the hypogonadal than in the eugonadal group (*p* < 0.05), whereas it was significantly lower in the treated hypogonadal vs. hypogonadal group (*p* < 0.05), showing no significant difference from the eugonadal group (Fig. 3, panel a). Worth noting, similar findings were observed when liver biopsies were analyzed for the steatosis score (Fig. 3, panel b). Furthermore, considering cFT quartiles measured at V1 (pre-surgery visit), we observed significant negative age-adjusted correlations with steatosis (*r*: − 0.362, *p* < 0.01) and inflammation scores (*r*: − 0.438, *p* < 0.05).

**Fig. 4:**
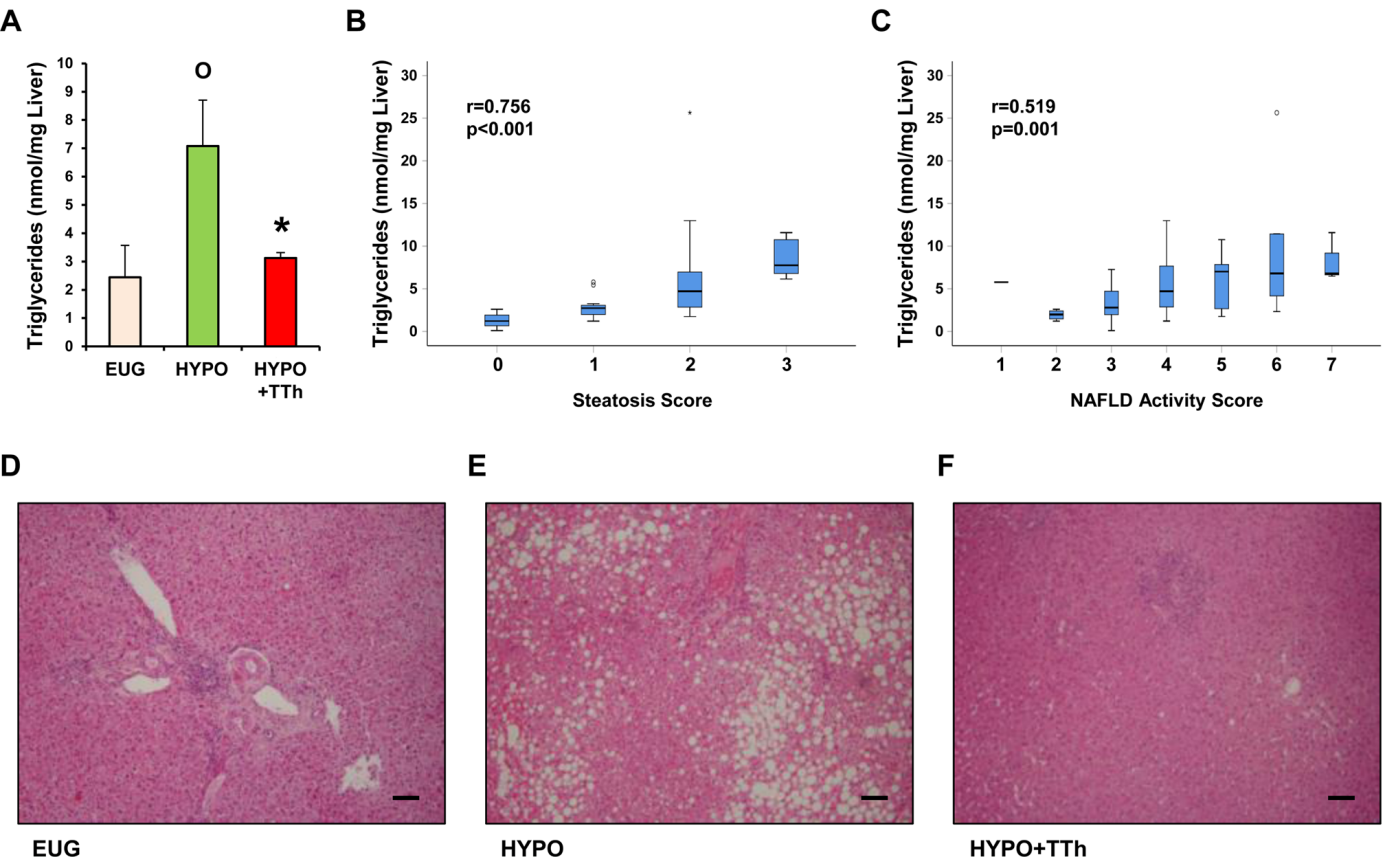
**a** Bar graph shows liver triglyceride levels obtained from liver biopsies in all three groups of patients. **b**, **c** Display the correlation of liver triglyceride levels with steatosis and NAS scores, respectively. **d**–**f** Show the H&E staining of liver sections from eugonadal (EUG), hypogonadal (HYPO) and hypogonadal subjects treated with testosterone (HYPO + TTh), respectively. ^**O**^*p* < 0.05 vs. Eugonadal; *p < 0.05 vs. Hypogonadal; Scale bar 100 μm

Intrahepatic triglyceride (TG) levels obtained from liver biopsies collected during surgical procedure resulted in being significantly higher in the hypogonadal group, when compared to eugonadal subjects (Fig. 4 panel a; *p* < 0.05). Interestingly, T-treated hypogonadal subjects showed significantly reduced intrahepatic TG level, with respect to untreated-hypogonadal subjects (Fig. 4 panel a; *p* < 0.05 vs. hypogonadal), reaching a level that was almost identical to that observed in eugonadal patients. Considering all groups, intrahepatic TG levels correlated positively with both steatosis (Fig. 4 panel b; *p* < 0.001) and NAS scores (Fig. 4 panel c; *p* < 0.001). Hepatic histomorphological analysis, performed using H&E staining, demonstrated that hypogonadism was associated with a prominent lipid accumulation within the hepatocytes, compared to eugonadal patients (Fig. 4 panels d, e, respectively). In contrast, macrovesicular steatosis was dramatically reduced in testosterone-treated hypogonadal subjects (Fig. 4 panel f).

### Treatment with testosterone enhances the expression of genes involved in lipid metabolism and insulin signaling in liver tissue

**Fig. 5:**
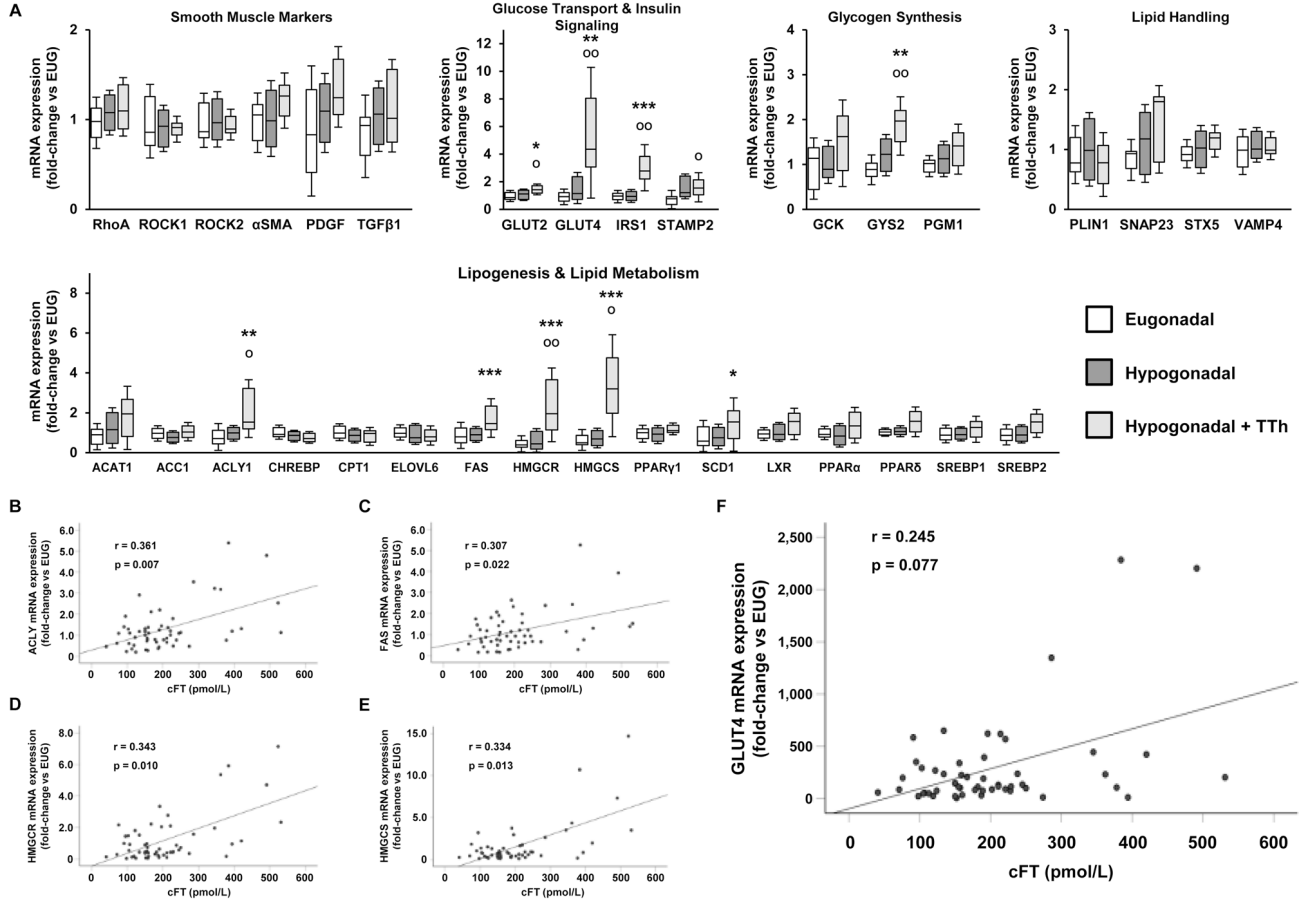
**a** Displays the liver tissue relative mRNA expression of smooth muscle/fibrosis markers and genes related to glucose transport, insulin signaling, glycogenesis and lipid handling and metabolism. Data are calculated per the 2^−ΔΔCt^ comparative method, using the 18S ribosomal RNA subunit as the reference gene for normalization. Results are expressed as fold-change vs. the eugonadal group and are reported in a box plot as interquartiles ± SEM. Statistical analysis was performed using Kruskal–Wallis and Mann–Whitney tests. **b**–**f** show the correlations between cFT and ACLY, FAS, HMGCR, HMGCS and GLUT4 liver mRNA expression, respectively. (Eugonadal, *n* = 15; Hypogonadal, *n* = 26; Hypogonadal + TTh, *n* = 15). ^**O**^*p* < 0.05, ^**OO**^*p* < 0.01 vs. Eugonadal; *p < 0.05, **p < 0.01, ***p < 0.001 vs. Hypogonadal

In the liver, testosterone treatment resulted in an increased mRNA expression of several genes involved in hepatic lipid and glucose/glycogen metabolism (Fig. 5 panel a). A significant increase was observed for mRNA expression of key enzymes related to the balance between hepatic lipid accumulation and β-oxidation, such as FAS, or related to ketogenesis, including HMGCS. The lipotoxicity protective factor, SCD1, and other enzymes related to lipid metabolism, such as ACLY and HMGCR, were upregulated in TTh-hypogonadal subjects (Fig. 5 panel a). Interestingly, mRNA expression of most of these enzymes correlated positively with cFT at pre-surgery V1 (Fig. 5 panels b–e). In the liver, a clear increase trend in the mRNA expression of key enzymes involved in glycogen synthesis was induced by TTh, in particular with significant upregulation of GYS2 mRNA (Fig. 5 panel a). Likewise, the mRNA expression of genes involved in glucose transport and insulin signaling (GLUT2, GLUT4, IRS1 and STAMP2) was also significantly increased by TTh (Fig. 5 panel a), with GLUT4 liver mRNA expression showing a trend for positive correlation with cFT, although without reaching statistical significance (Fig. 5 panel f; *p* = 0.077). We also found a close positive correlation between AR and the mRNA expression of several liver genes that participate in the regulation of lipid and glucose metabolism (Table 4).

**Table 4:** Association between AR mRNA and the expression of target genes related to lipid metabolism, lipid handling, glycogen synthesis, glucose transport and insulin-signaling in the liver

AR mRNA expression	*r*	*p*
Lipid metabolism-related genes		
ACAT1	0.812	0.000
ACC1	0.271	0.043
ACLY1	0.674	0.000
ChREBP	− 0.291	0.030
CPT1	0.028	0.837
ELOVL6	0.138	0.309
FAS	0.440	0.001
HMGCR	0.656	0.000
HMGCS	0.643	0.000
LXR	0.742	0.000
PPARα	0.792	0.000
PPARδ	0.722	0.000
PPARγ	0.660	0.000
SCD1	0.612	0.000
SREBF1	0.589	0.000
SREBF2	0.692	0.000
Lipid handling-related genes		
PLIN	0.398	0.002
SNAP23	0.673	0.000
STX5	0.708	0.000
VAMP4	0.350	0.009
Glycogen synthesis-related genes		
GCK	0.145	0.338
GYS2	0.420	0.003
PGM1	0.637	0.000
Glucose Transport- and Insulin signaling-related genes		
GLUT2	0.611	0.000
GLUT4	0.660	0.000
IRS1	0.522	0.000
STAMP2	0.288	0.035

Correlations coefficients (*r*) and level of significance (*p*) are derived from univariate analysis

### Effects of testosterone treatment on the expression of genes involved in brown adipogenesis, lipid catabolism and mitochondrial bio-functionality in visceral fat tissue

**Fig. 6:**
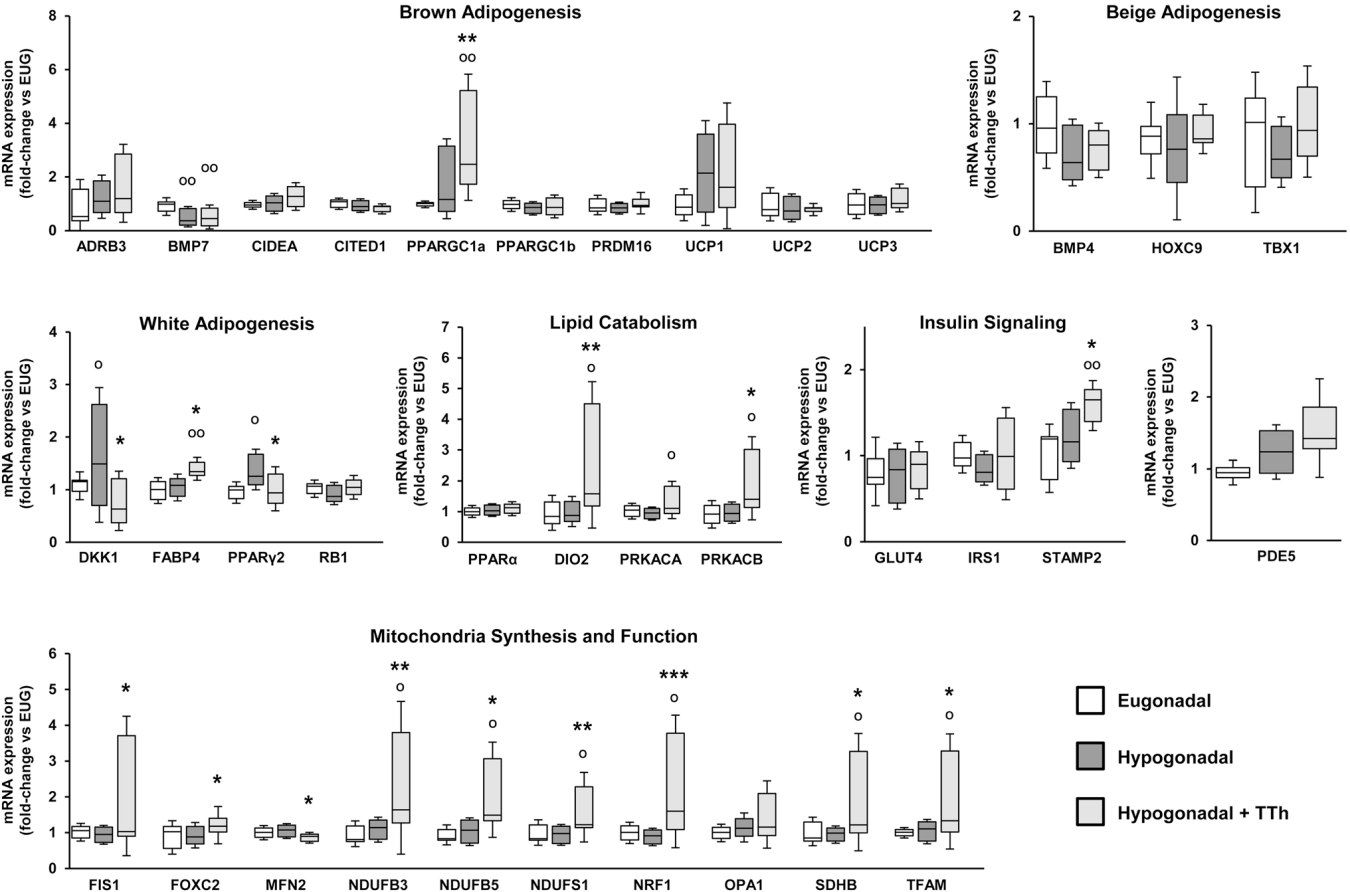
Visceral adipose tissue relative mRNA expression of genes related to brown, beige and white adipogenesis, lipid catabolism, insulin signaling and mitochondrial life cycle. Data are calculated per the 2^−ΔΔCt^ comparative method, using the 18S ribosomal RNA subunit as the reference gene for normalization. Results are expressed as fold-change vs. the eugonadal group and are reported in a box plot as interquartiles ± SEM. Statistical analysis was performed using Kruskal–Wallis and Mann–Whitney tests. (Eugonadal, *n* = 15; Hypogonadal, *n* = 37; Hypogonadal + TTh, *n* = 15). ^**O**^*p* < 0.05, ^**OO**^*p* < 0.01 vs. Eugonadal; *p < 0.05, **p < 0.01, ***p < 0.001 vs. Hypogonadal

When visceral fat tissue biopsies obtained at surgery were examined, we observed that TTh was associated with a sharp increase of the mRNA expression of several genes involved in mitochondrial biogenesis (NRF1, TFAM) and function (FIS1, FOXC2, NDUFB3, NDUFB5, NDUFS1, SDHB), when compared to both untreated hypogonadal and eugonadal subjects (Fig. 6). Additionally, when compared to hypogonadal patients, TTh was also associated with an increased mRNA expression of brown adipogenesis marker PPARGC1a (*p* < 0.01). Accordingly, master regulators of white adipogenesis, namely PPARγ2 and DKK1, which were significantly increased in untreated-hypogonadal subjects, were normalized in T-treated hypogonadal subjects (Fig. 6; *p* < 0.05). Also lipid catabolism markers such as DIO2 (*p* < 0.01), PRKACA (*p* < 0.05) and PRKACB (*p* < 0.05) were significantly increased in TTh-hypogonadal patients (Fig. 6). The mRNA expression of PDE5 did not show statistically significant differences among the three groups (Fig. 6). We observed a very moderate increase in the mRNA expression of inflammation markers (COX2, GATA3, IL6, IL8, IL12, LOX1, MCP1, RORγT, TBET) in visceral adipose tissue from hypogonadal patients, a phenomenon that was not affected by TTh (data not shown). Finally, T treatment was associated with a significantly higher mRNA expression of STAMP2, when compared to hypogonadal and eugonadal subjects (Fig. 6).

### Effects of testosterone treatment on the expression of genes involved in lipid metabolism and mitochondrial bio-functionality in hPADs

**Fig. 7:**
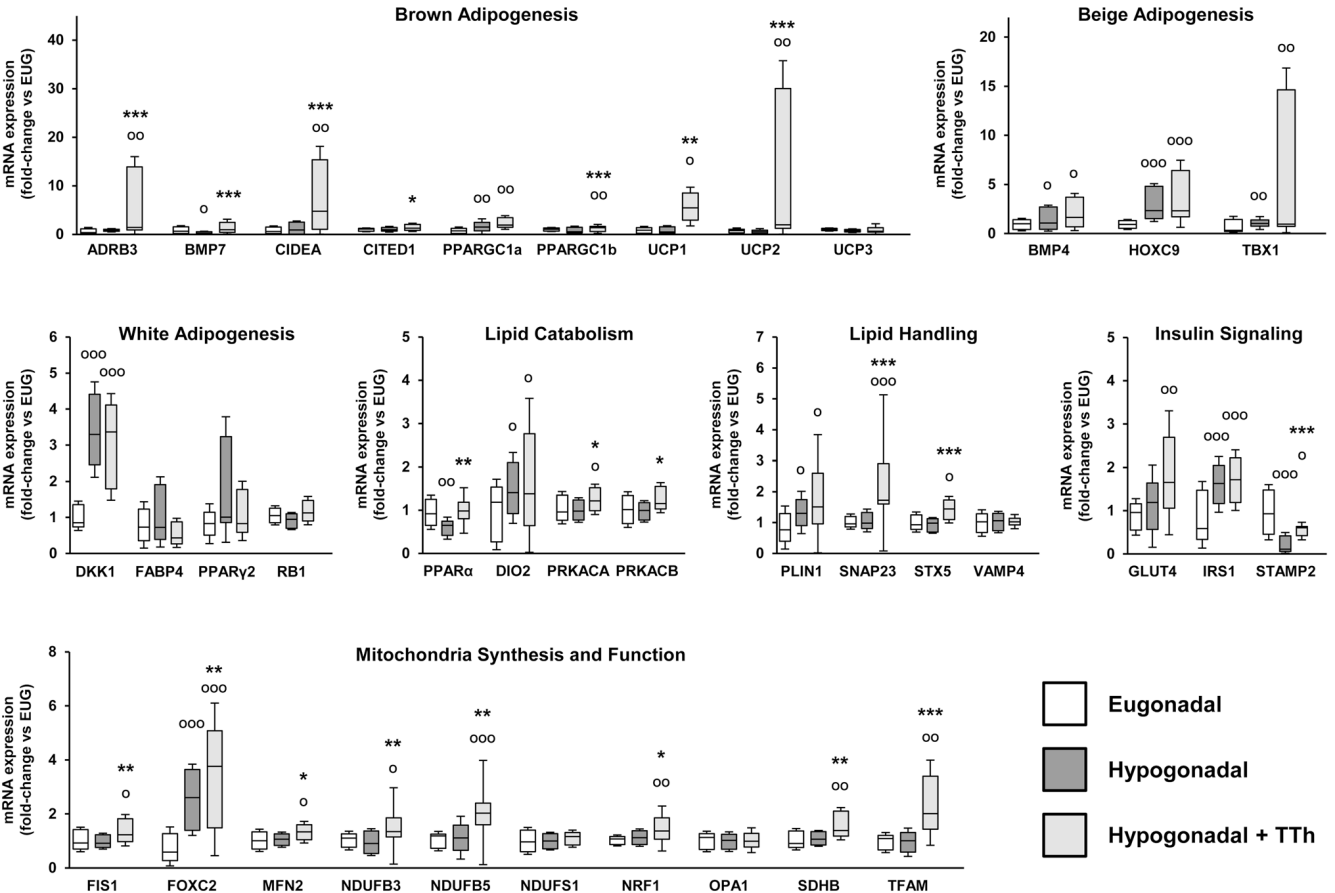
Human preadipocytes (hPADs) relative mRNA expression of genes related to brown, beige and white adipogenesis, lipid catabolism and handling, insulin signaling and mitochondrial life cycle. Data are calculated per the 2^−ΔΔCt^ comparative method, using the 18S ribosomal RNA subunit as the reference gene for normalization. Results are expressed as fold-change vs. the eugonadal group and are reported in a box plot as interquartiles ± SEM. Experiments were performed in triplicate using four different hPADs preparations. Statistical analysis was performed using Kruskal–Wallis and Mann–Whitney tests. ^**O**^*p* < 0.05, ^**OO**^*p* < 0.01, ^**OOO**^*p* < 0.001 vs. Eugonadal; *p < 0.05, **p < 0.01, ***p < 0.001 vs. Hypogonadal

We next examined the DIM-induced differentiation of human preadipocytes (hPADs) isolated from the different groups after 10 days of culture. Compared to hPADs obtained from eugonadal patients, hPADs from untreated-hypogonadal patients showed a significant reduction of the mRNA expression of specific markers of brown adipogenesis (BMP7), lipid catabolism (PPARα) and insulin signaling (STAMP2) (Fig. 7; *p* < 0.05) with a parallel increase of genes related to beige/white adipogenesis (BMP4, HOXC9, TBX1; DKK1, FABP4), with PPARγ2 showing an evident, albeit not significant, increase that was normalized by TTh (Fig. 7). On the contrary, in hPADs from T-treated subjects we found a significant increase in the mRNA expression of several genes involved in brown adipogenesis (ADRB3, BMP7, CIDEA, CITED1, UCP1, UCP2), lipid catabolism (PPARα, PRKACA, PRKACB), lipid handling (SNAP23, STX5) and mitochondrial biosynthesis and function (FIS1, FOXC2, MFN2, NDUFB3, NDUFB5, NRF1, SDHB, TFAM), when compared with hPADs derived from hypogonadal patients. The mRNA expression of these genes, in hPADs originating from the T treatment group, resulted often even significantly higher than the levels observed in hPADs derived from the eugonadal group (Fig. 7).

### Testosterone enhances insulin sensitivity in DIM-induced preadipocytes

**Fig. 8:**
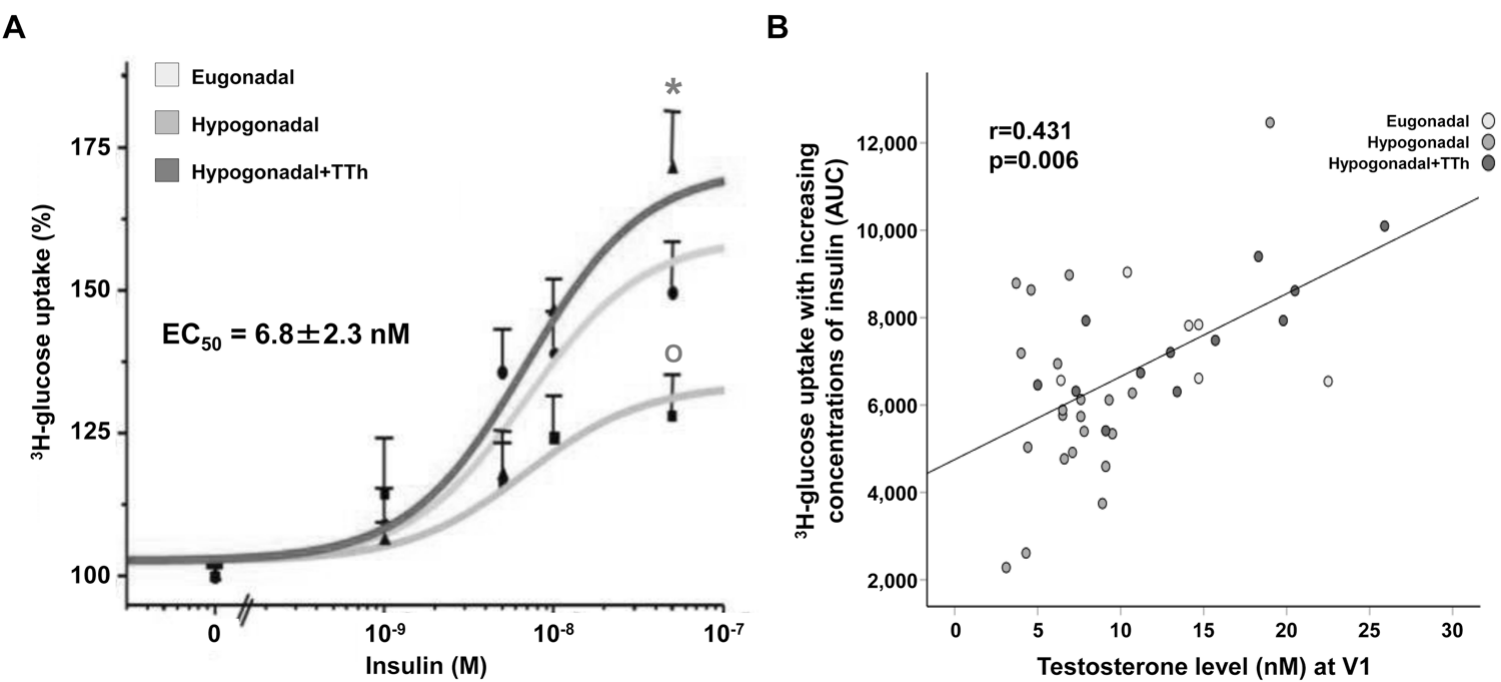
Panel A shows the insulin dose-dependent ^3^H-2-deoxy-d-glucose uptake in hPADs from all groups after exposure (30 min) to increasing concentrations of insulin. Results are expressed in percentage over baseline (no insulin) and are reported as mean ± SEM of four different experiments, each performed in duplicate and using a different cell preparation per group. **b** Shows the glucose uptake AUC correlation with total testosterone levels at pre-surgery V1 [AUC: incremental area under the curve of glucose blood level during oral glucose tolerance test (OGTT)]. ^**O**^*p* < 0.05 vs. Eugonadal; *p < 0.05 vs. Hypogonadal

In vivo testosterone treatment enhanced the insulin-induced uptake of ^3^H-2-deoxy-d-glucose in hPADs. In DIM-induced hPADs from all groups, insulin increased ^3^H-2-deoxy-d-glucose uptake in a dose-dependent manner, with similar EC_50_ values (shared EC_50_ = 6.8 ± 2.3 nM), but with different maximal effects (Fig. 8 panel a). Indeed, in hPADs from hypogonadal patients, insulin E_max_ was significantly reduced compared to eugonadal hPADs (Fig. 8 panel a; *p* < 0.05). Treatment with T normalized the ability of hPADs to respond to increasing concentrations of insulin (Fig. 8 panel a; *p* < 0.05 vs. hypogonadal). Accordingly, the glucose uptake AUC showed a positive correlation with testosterone levels (Fig. 8 panel b; *r* = 0.431; *p* = 0.006).

### Treatment with testosterone enhances mitochondrial function and preserves their ultrastructure

**Fig. 9:**
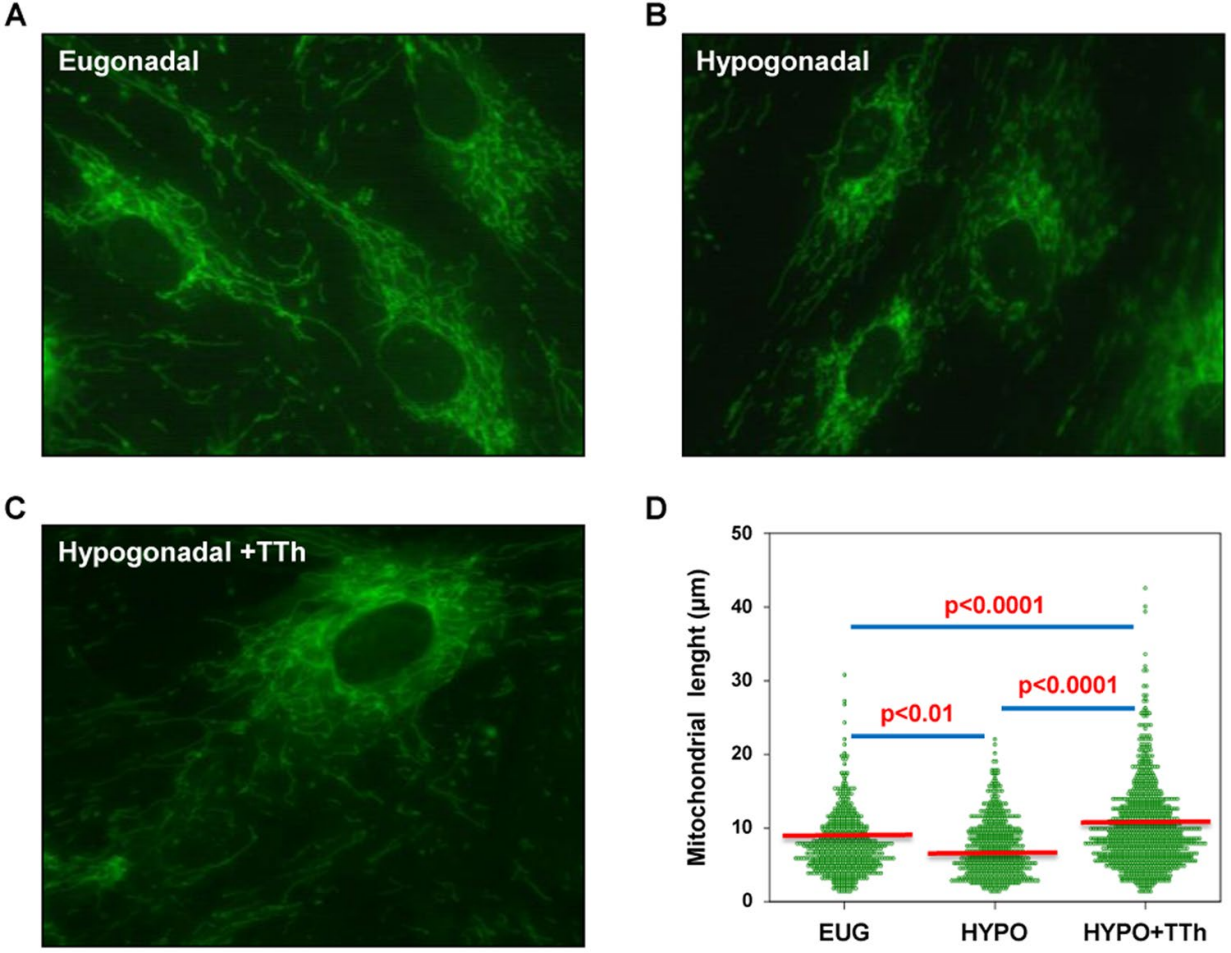
**a**–**c** Show representative time-lapse images of the mitochondrial function in hPADs isolated from all groups of patients, visualized by incubation of the mitochondria-targeted fluorescent probe (MitoTracker staining) and imaged for 3 min. Computer-assisted measurement of mitochondria length is reported in **d**, and respective *p* values are reported within the panel. For morphometric analysis of mitochondrial length, well-resolved mitochondria in the cell periphery were analyzed by ImageJ software. At least 50 individual mitochondrial structures in at least 10 cells/group were measured to determine mitochondrial length (μm) distribution. Data were obtained from three independent experiments

To address the mechanisms underlying testosterone-induced effects, we studied the mitochondrial function in hPADs using MitoTracker, a mitochondria-targeted fluorescent probe. Cells with similar shapes were chosen and time-lapse microscopic imaging was used to assess mitochondrial dynamics (Fig. 9 panels a–c). Computer-assisted measurement of mitochondria length in hPADs is reported in Fig. 9 panel d. Mitochondria in eugonadal hPADs showed heterogeneity in shape and length, with small and elongating mitochondria (Fig. 9 panel a), resulting in a dynamic behavior and a wide length distribution (Fig. 9 panel d). In hPADs from hypogonadal patients, mitochondria appeared randomly dispersed, fragmented (Fig. 9 panel b) and immotile, with less elongated mitochondrial network and a lower average length (Fig. 9 panel d; *p* < 0.01 vs. eugonadal). Interestingly, the mitochondrial networks of hPADs from the T-treated group were continuously undergoing mitochondrial fission (fragmentation or division) and fusion (rejoining), with rapid changing in shape and length (Fig. 9 panel c), with an increased average length compared to both hypogonadal and eugonadal samples (Fig. 9 panel d; both *p* < 0.0001).

### Effects of testosterone treatment on mitochondrial superoxide generation and oxygen consumption

**Fig. 10:**
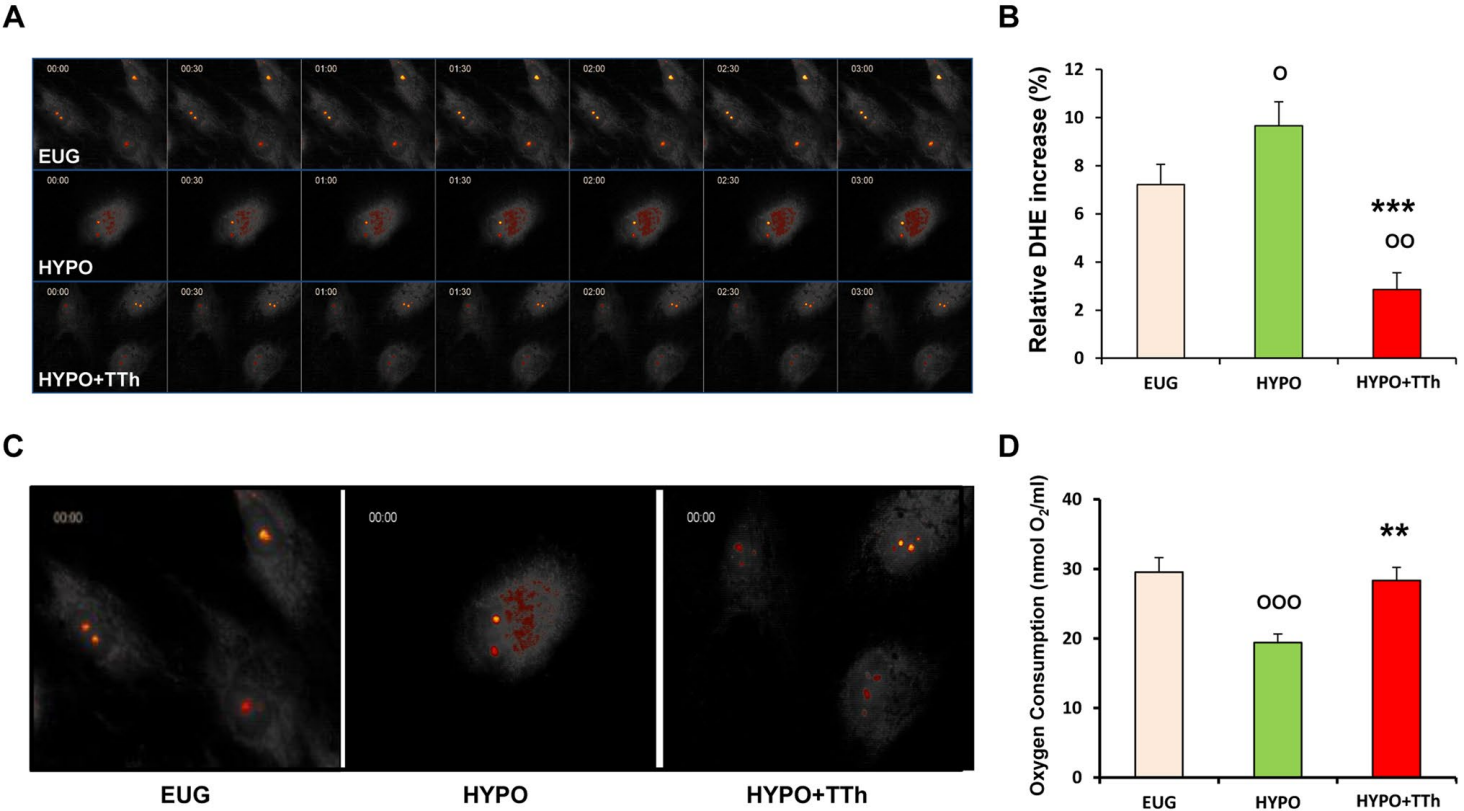
**a** Displays representative time-lapse images of hPADs isolated from EUG, HYPO and HYPO + TTh patients, stained with 10 μM dihydroethidium (DHE) and imaged for 3 min. **b** Bar graph shows the changes in integrated fluorescence intensity measured in the nuclei of hPADs during time-lapse imaging. **c** Shows a higher magnification of DHE-derived fluorescence in each group. Panel D shows the oxygen consumption in hPADs isolated from EUG, HYPO and HYPO + TTh patients after 10 days of spontaneous differentiation. It was measured by the Oxygraph system instrument. The bar graph shows the ratio of oxygen consumption normalized per mL of cell volume. Data are reported as the mean ± SEM. of at least three independent experiments. ^**O**^*p* < 0.05, ^**OO**^*p* < 0.01, ^**OOO**^*p* < 0.001 vs. Eugonadal; **p < 0.01, ***p < 0.001 vs. Hypogonadal

Time-dependent accumulation of dihydroethidium (DHE)-derived fluorescence was studied as a surrogate marker of spontaneous superoxide production in hPADs (Fig. 10 panel a). Eugonadal hPADs did not show any DHE-derived fluorescence accumulation over time (sequential 30 s images; Fig. 10 panel a). Conversely, a time-dependent evident increase in superoxide generation was observed in hPADs from hypogonadal patients (sequential 30 s images; Fig. 10 panel a), whilst T treatment markedly reduced superoxide production (sequential 30 s images; Fig. 10 panel a). Quantification of fluorescence intensity changes over time (Fig. 10 panel b) also indicated that T treatment significantly reduced superoxide accumulation observed in untreated-hypogonadal subjects, even significantly below eugonadal level. Figure 10 panel c shows a higher magnification of DHE-derived fluorescence in each group.

To assess the mitochondrial activity, we next quantified oxygen consumption in hPADs isolated from each experimental group. The hypogonadal group showed a significant reduction (Fig. 10 panel d; *p* < 0.05) of oxygen consumption compared to eugonadal subjects, whereas testosterone treatment significantly increased the O_2_ consumption levels, compared to hypogonadal patients (*p* < 0.05), restoring the values up to eugonadal levels (Fig. 10 panel d).

## Discussion

The study is the first to demonstrate that in vivo treatment with the long-lasting T preparation T undecanoate in symptomatic hypogonadal obese patients is associated with less severe NASH and visceral adipose tissue dysfunction as compared to untreated-hypogonadal ones. Liver biopsies from T-treated hypogonadal patients showed significantly lower intrahepatic triglyceride content, macrovesicular lipid accumulation and NAS score for steatosis, as compared to untreated-hypogonadal subjects. Accordingly, the clinical and biochemical algorithm which reflects fatty liver was also significantly reduced in TTh-treated hypogonadal patients over time, as compared to untreated-hypogonadal ones. However, the most remarkable results emerge from the experiments in differentiating preadipocytes isolated from human visceral adipose tissue samples of the different treatment groups (eugonadal, TTh-hypogonadal and untreated-hypogonadal subjects). In this large set of experiments, we demonstrated that preadipocytes from TTh patients showed a metabolically healthy phenotype as evidenced by a significantly healthier mitochondrial function, along with a higher insulin sensitivity and oxygen consumption paralleled by a lower superoxide accumulation. Preadipocytes from TTh-hypogonadal subjects also showed a higher expression of all the genes related to mitochondrial function, beige-brown adipogenesis, insulin signaling, and lipid metabolism, as compared to those from untreated-hypogonadal ones. These cross-sectional data derived from liver and fat biopsies were substantiated further by prospective data on biochemical and metabolic parameters. TTh significantly reduced serum cholesterol levels over time (pre-surgery vs. baseline visit), whilst a significant increase with time of triglycerides and transaminases along with a decrease in insulin serum levels (despite higher fasting glucose) was observed in untreated, but not in TTh-treated, hypogonadal subjects. Therefore, our findings further support pre-existing high-quality evidence that TTh induced a range of beneficial metabolic effects in hypogonadal patients [49]. In a recent study by Di Nisio and colleagues [50], a significant deregulation of gene responsiveness to testosterone in obese subcutaneous adipose tissue (SAT) was observed, with testosterone accumulation resulting in lower expression of genes involved in lipolytic and anti-adipogenic pathways, thus suggesting an altered response of dysfunctional fat cells already present in mild obesity. These data corroborate our finding relative to VAT and preadipocytes derived from class III obesity subjects, in which hypogonadism was associated with a decreased expression of genes involved in brown adipogenesis and lipid catabolism. Consistent with these positive metabolic effects of testosterone, a recent meta-analysis of RCTs demonstrated that TTh significantly reduced fasting glycaemia, HOMA index, and fat mass, while increasing lean mass, as compared to placebo [49]. A nonsignificant trend toward reduced total cholesterol and triglyceride level was also induced by TTh [49]. In accordance, a meta-analytic evaluation of observational studies demonstrated that TTh also improves glyco-metabolic control (reduction of HOMA, fasting glycaemia), lipid profile (reduction of triglycerides and total cholesterol level and increased HDL), and body composition (reduction of waist circumference, BMI, body weight and fat mass, and increased lean mass) [27]. A relevant study on a similar population of severely obese hypogonadal men reported an improvement in several cardio-metabolic parameters after 1-year TTh, with only some retained after 24 weeks from withdrawal (i.e. fat, but not lean mass amelioration, was maintained) [51]. Despite these clinically relevant findings, there is limited evidence on the putative biological and molecular mechanisms underlying TTh metabolic effects. In this context, the key advantage of the present study design is that it allowed us to explore, not only the systemic, but also the direct, local (within liver and adipose tissue) impact of TTh in severely obese patients, by analyzing and comparing tissue samples collected during the surgical procedure from the three different study arms (eugonadal, T-untreated hypogonadal and T-treated hypogonadal). Interestingly, from adipose tissue samples, we were also able to isolate and differentiate preadipocytes, whose dysfunction has a central role in driving insulin resistance [52]. One of the most intriguing findings was that preadipocytes isolated from TTh subjects showed significantly improved mitochondrial morpho-functional features, as compared to those form untreated- hypogonadal subjects. Mitochondria dysfunctions are nowadays considered as the major underlying driver of several metabolic diseases [53, 54]. Mitochondria constantly undergo fusion and division processes to form a tubular network, which is crucial for health in most eukaryotic cells [55, 56]. In unhealthy cells, division (fission) becomes predominant and the mitochondrial network fragments, thus reducing energy efficiency, ATP production and fitness to respond to environmental stress buffering reactive oxygen species (ROS) [57–62]. Accordingly, mitochondria in differentiating preadipocytes from untreated-hypogonadal subjects were small, immobile and highly fragmented with reduced networking, oxygen consumption and increased superoxide formation. In contrast, preadipocytes from T-treated hypogonadal patients showed increased mitochondria dynamics and interconnectivity, coupled with increased oxygen consumption and reduced superoxide content. A significantly higher mRNA expression level of mitochondrial biogenesis (NRF1, TFAM), networking (MFN2, FIS1) and function (NDUFB3, NDFUB5, SDHB, FOXC2) markers was also observed in preadipocytes from TTh-hypogonadal patients as compared to untreated-hypogonadal ones. Interestingly, in a rodent model, synergistic activation of two of these markers, namely TFAM and FOXC2, enhanced adipose tissue expression of core proteins of mitochondrial fusion (MFN1, MFN2, and OPA1) consequently leading to a lean and insulin-sensitive phenotype [63–66]. Noteworthy, given that TFAM, one of the most important mitochondrial DNA transcription factors [67–69], contains AR-responsive elements, it is now considered a relevant AR target gene through which testosterone might regulate mitochondrial homeostasis [70]. In line with this evidence, a recent study demonstrated that AR, besides being nuclear, also localizes into mitochondria, where it modulates some of the non-genomic effects of androgens [71]. Therefore, our data further corroborated the positive effects of T on mitochondrial function. A marked increase of genes related to lipid metabolism/handling (including PPARα, PRKACA, PRAKCB, SNAP23, STX5) and reactive oxygen species removal (such as PPARGC1β) were also observed in preadipocytes from T-treated hypogonadal patients. Lipid handling and lipid droplet formation are closely related to mitochondria processes [61]. Essentially, a direct delivery of lipids from lipid droplets into mitochondria represents an efficient way to maintain ROS within physiological levels, thus shielding other cell organelles from lipotoxicity, while ensuring energy supply as well as insulin sensitivity [11, 61, 72, 73]. Consistent with this view, we observed a reduced superoxide formation, which was associated with an increase of oxygen consumption and insulin sensitivity in preadipocytes after i*n vivo* TTh. In particular, these preadipocytes demonstrated a significantly improved insulin-stimulated glucose uptake, as compared to preadipocytes from untreated-hypogonadal obese subjects, reaching a level that was even higher than that of eugonadal ones. Noteworthy, the ability of preadipocytes to respond to increasing concentrations of insulin was tightly dependent upon T level, measured at the pre-surgery visit 1. The improved metabolic functions of preadipocytes from TTh patients were further supported by a significant increase of all the genes related not only to insulin signaling, mitochondrial biogenesis and lipid handling, but also to brown/beige adipogenesis (ADRB3, CIDEA, CITED1, BMP4, BMP7, HOXC9, TBX1) [74, 75]. In particular, a marked induction of UCP1 and UCP2 was observed in PADs derived from adipose tissue of T-treated hypogonadal obese men. The development of brown/beige adipocytes can be induced in white adipose tissue (WAT), because of a sustained stimulation with cold, ß3-adrenergic (ADRB3), peroxisome proliferator-activated receptor γ (PPARγ) agonists, or BMP7. This program is accompanied by induction of uncoupling proteins, especially UCP1, along with an elevated mitochondrial respiration and thermogenesis [76, 77]. In our study, we also found a significant and huge increase of the UCP isoform 2 (UCP2). Interestingly, UCP2 is an ion/anion transporter located in the mitochondrial inner membrane, with a central function in the regulation of oxidative stress, cellular metabolism and whole energy homeostasis [78]. A reduced UCP2 expression and activity has been linked to obesity-associated metabolic disorders, such as T2DM, hypertension and atherosclerosis [79–82]. In hPADs collected from TTh men, we also found a concomitant increase of crucial factors (e.g. DKK1, DIO2) for adipose tissue differentiation and the maintenance of adipocyte homeostasis, which allows for the balance between lipolysis and adipogenesis [83]. The mRNA expression of PDE5 in adipose tissue, where it is known to be abundantly expressed [84], did not show significant changes. Finally, the observed increase of UCP1 mRNA expression in VAT from both untreated and treated hypogonadal patients, although not significant, could represent an attempt to counterbalance the potentially reduced UCP1 activity in the hypogonadal phenotype. This phenomenon would find its major efficacy in isolated preadipocytes, which display a significant UCP1 mRNA expression increase only in the TTh group, although the evaluation of UCP1 phosphorylation, rather than mRNA expression, would provide more useful information with regard to its activity. These observations would merit to be analyzed in further studies, perhaps including specific researches on enzymatic activity in a similar model setting.

The aforementioned observations depict a metabolically healthier phenotype for hPADs collected from T-treated obese hypogonadal subjects, as compared with untreated-hypogonadal men. Our data also corroborated the pivotal role of T in maintaining the homeostasis of mitochondria, the most energetically active organelles in the eukaryotic cells [85]. Previous studies, both in animal models and in humans, demonstrated that androgens stimulate glucose utilization and ATP production, as well as mitochondrial cytochrome c oxidase activity in muscle [86–88], and in several other tissues and cell types [89–92]. However, to our knowledge, this is the first demonstration that in vivo TTh is associated with a healthier phenotype of human preadipocytes. Accordingly, a significantly increased expression of several genes related to mitochondria biogenesis and function, as well as brown/beige adipogenesis, was also observed in the homogenates of visceral adipose tissue samples from TTh-hypogonadal obese subjects, as compared to those of eugonadal and untreated-hypogonadal ones.

Another striking observation of our study is that untreated hypogonadism was associated with a three-fold higher intrahepatic level of triglycerides (assessed in liver biopsies), as compared to eugonadism. This marked increase was not observed in TTh-subjects, whose levels were comparable to eugonadal ones. It is noteworthy that intrahepatic triglyceride content was closely correlated to the histological score of steatosis and with the NAFLD activity score. The fact that we did not observe a parallel decrease in triglycerides levels in TTh-subjects suggests that liver changes could be considered not as the consequence of lower circulating triglycerides, but indeed as the *primum movens* of the metabolic actions of TTh. As a result, the reduction in hepatic steatosis may be detectable before its consequences appear systemically. Importantly, TTh was also associated with an increased liver expression of all the genes related to insulin signaling, such as GLUT4, IRS1, STAMP2 [93–95], insulin-independent glucose transport (GLUT2) and genes related to glycogen synthesis (of note, glycogen synthase 2, GYS2), and intrahepatic lipid handling. The data relative to the increased expression of GYS2 are substantiated by previous results showing that testosterone replacement favored glycogen synthesis in the skeletal muscle in animal models [96]. Liver from TTh-hypogonadal subjects showed a significant upregulation of FAS, the rate-limiting enzyme in the fatty acid biosynthesis and HMGCs, a catalytic enzyme of ketogenesis that provides lipid-derived energy. Noteworthy, the liver specific FAS knockout aggravates hepatic steatosis, thus suggesting that FAS has a prominent protective effect in the liver [97, 98].

SCD1, an enzyme catalyzing the synthesis of oleate, was also increased in the TTh group, as compared to the untreated one. SCD1 is involved in carbohydrate-induced activation of FAS and other lipogenic enzymes, and recently proposed as a potential therapeutic target to control obesity and the progression of related metabolic diseases including hepatic steatosis [99]. Therefore, the SCD1 mRNA increase in a context of histologically- and biochemically-proven intrahepatic lipid reduction, as observed in T-treated subjects, should be interpreted cautiously. It is plausible that its mRNA increase, in this context, represents a compensatory mechanism in response to a general reduction of liver lipogenesis, rather than a marker of an increased fatty acid formation occurring upon TTh. In line with this interpretation, mRNA expression level of several genes related to liver lipid handling, metabolism and β-oxidation showed significant positive correlations with calculated free T, as assessed at the pre-surgery visit. The vast majority of these genes were also closely associated with AR mRNA expression. More importantly, histological total NAS score, as well as the subscores related to steatosis and inflammation within the liver, decreased as a function of calculated free T level at the pre-surgery visit. TTh-hypogonadal subjects showed also a significant reduction of fatty liver index (FLI) and total cholesterol level overtime. In particular, fatty liver index (FLI)—calculated by a specific algorithm based on BMI, waist circumference, triglycerides and yGT—is recognized as an accurate predicting parameter of histologically proven fatty liver [43]. Therefore, given that our main findings are based on differences among treatment groups as assessed within surgical samples, the significant change over time of FLI further strengthened the suggested beneficial effect of TTh on the liver. An association between lower levels of total T and the development of NAFLD has already been described in the clinical [100–102] and preclinical setting [31, 103–105]. A significant improvement of non-alcoholic liver steatosis upon treatment of hypogonadal elderly men with parenteral T undecanoate has already been reported [32, 33, 106]. However, having a liver biopsy collected from patients receiving TTh before the surgical procedure, which demonstrated a significant reduction of liver abnormalities, is one of the main strengths of our study.

In contrast to earlier findings [27], we did not find any significant differences between TTh and untreated-hypogonadal subjects in terms of BMI, or glyco-metabolic indices. These discrepancies might be attributed to the shorter therapy duration of our study, as compared to the previous observational trials [27]. Indeed Corona and co-workers found that the strength of the beneficial effects of TTh on weight increased as a function of trial duration. To make an example, body weight showed a significant reduction after 24 months of TTh, with an estimated loss of − 3.50 [− 5.21; − 1.80] kg [27]. Our observational study was designed to evaluate the effect of TTh based on the time interval spent on the waiting list before the patients received bariatric surgery (roughly 12 months). Another important limitation of the present study lies in the fact that it cannot establish causality due to its observational design. Therefore, it could be hypothesized that men in the eugonadal and hypogonadal (asymptomatic) untreated groups are healthier than the hypogonadal treated (symptomatic) patients. Hence, it could be hypothesized that men in the hypogonadal untreated group are intrinsically less exposed to the detrimental effects of T deficiency than the investigated treated group, made up of hypogonadal symptomatic patients. This establishes a possible selection bias. However, baseline value of body weight, waist circumference, glycol-lipid parameters and blood pressure of the three groups were not statistically different, therefore minimizing the risk of a baseline better cardio-metabolic profile in the TTh arm. Similarly, other possible confounders, including the prevalence of comorbidities (type 2 Diabetes Mellitus, hypertension, cardiovascular diseases), the use of glucose- and lipid-lowering drugs and of PDE5 inhibitors did not show a significant difference between the three groups. It should also be considered that liver and visceral fat biopsies were performed only at the time of bariatric surgery; therefore, data on hepatic inflammation, steatosis and triglyceride content do not allow for a comparison between post-treatment and baseline. This limitation, due to the invasiveness of tissue sample collection, is, at least partially, overcome by the use of the eugonadal group as the comparator for the analyses on these tissues and cells. Finally, a Magnetic Resonance Imaging (MRI) study of liver at baseline and after TTh or a FibroScan evaluation of hepatic tissue would have add interesting data.

In conclusion, we provide evidence that TTh in hypogonadal symptomatic severely obese patients is associated with a reduced preadipocytes dysfunction through an increase of insulin sensitivity, an improvement in mitochondrial function and lipid handling, eventually leading to their differentiation towards a metabolically healthier phenotype. Similarly, testosterone was associated with reduced hepatic steatosis and intrahepatic content of triglycerides. Randomized clinical trials are warranted to confirm these observations.
